# The evolution and multi-molecular properties of NF1 cutaneous neurofibromas originating from C-fiber sensory endings and terminal Schwann cells at normal sites of sensory terminations in the skin

**DOI:** 10.1371/journal.pone.0216527

**Published:** 2019-05-20

**Authors:** Frank L. Rice, George Houk, James P. Wymer, Sara J. C. Gosline, Justin Guinney, Jianqiang Wu, Nancy Ratner, Michael P. Jankowski, Salvo La Rosa, Marilyn Dockum, James R. Storey, Steven L. Carroll, Phillip J. Albrecht, Vincent M. Riccardi

**Affiliations:** 1 Integrated Tissue Dynamics LLC, Rensselaer, NY, United States of America; 2 Division of Health Sciences, State University of New York at Albany, Albany, NY, United States of America; 3 Department of Neurology, University of Florida, Gainsville, FL, United States of America; 4 Sage Bionetworks, Seattle, WA, United States of America; 5 Division of Experimental Hematology and Cancer Biology, Cancer and Blood Diseases Institute, Cincinnati Children's Hospital, University of Cincinnati, Cincinnati, OH, United States of America; 6 Department of Pediatrics, Cincinnati Children’s Hospital Medical Center, University of Cincinnati, Cincinnati, OH, United States of America; 7 Department of Anesthesia, Division of Pain Management, Cincinnati Children’s Hospital Medical Center, University of Cincinnati, Cincinnati, OH, United States of America; 8 Children’s Tumor Foundation, New York, NY, United States of America; 9 Department of Neurology, Albany Medical College, Albany, NY, United States of America; 10 Department of Pathology and Laboratory Medicine, Medical University of South Carolina, Charleston, SC, United States of America; 11 The Neurofibromatosis Institute, La Crescenta, CA, United States of America; University of Würzburg, GERMANY

## Abstract

In addition to large plexiform neurofibromas (pNF), NF1 patients are frequently disfigured by cutaneous neurofibromas (cNF) and are often afflicted with chronic pain and itch even from seemingly normal skin areas. Both pNFs and cNF consist primarily of benign hyperproliferating nonmyelinating Schwann cells (nSC). While pNF clearly arise within deep nerves and plexuses, the role of cutaneous innervation in the origin of cNF and in chronic itch and pain is unknown. First, we conducted a comprehensive, multi-molecular, immunofluorescence (IF) analyses on 3mm punch biopsies from three separate locations in normal appearing, cNF-free skin in 19 NF1 patients and skin of 16 normal subjects. At least one biopsy in 17 NF1 patients had previously undescribed micro-lesions consisting of a small, dense cluster of nonpeptidergic C-fiber endings and the affiliated nSC consistently adjoining adnexal structures—dermal papillae, hair follicles, sweat glands, sweat ducts, and arterioles—where C-fiber endings normally terminate. Similar micro-lesions were detected in hind paw skin of mice with conditionally-induced SC *Nf1*^*-/-*^ mutations. Hypothesizing that these microlesions were pre-cNF origins of cNF, we subsequently analyzed numerous overt, small cNF (s-cNF, 3–6 mm) and discovered that each had an adnexal structure at the epicenter of vastly increased nonpeptidergic C-fiber terminals, accompanied by excessive nSC. The IF and functional genomics assays indicated that neurturin (NTRN) and artemin (ARTN) signaling through cRET kinase and GFRα2 and GFRα3 co-receptors on the aberrant C-fiber endings and nSC may mutually promote the onset of pre-cNF and their evolution to s-cNF. Moreover, TrpA1 and TrpV1 receptors may, respectively, mediate symptoms of chronic itch and pain. These newly discovered molecular characteristics might be targeted to suppress the development of cNF and to treat chronic itch and pain symptoms in NF1 patients.

## Introduction

Multiple cutaneous neurofibromas (cNF) are characteristic of neurofibromatosis type 1 (NF1) patients who have an autosomal dominant loss-of-function mutation of an *NF1* allele. cNF are visibly protruding masses in the skin composed of a complex mixture of hyperproliferating Schwann cells (SC), particularly nonmyelinating SC (nSC), intermingled with fibroblasts, vasculature, macrophages, mast cells and other cellular components [1–5]. This composition is similar to another hallmark of NF1, which are the expansive growths within large nerves and plexuses referred to as plexiform neurofibromas (pNF).

Although their histologic and genomic abnormalities are similar [1, 3, 6], the natural history of cNF and pNF is quite distinct. While pNF often present as congenital lesions, cNF are typically not present at birth [1, 2]. Instead, cNF usually begin to appear at puberty, accumulating thereafter to a variable degree over the remainder of the patient’s life. In addition, whereas pNF occasionally transform into malignant peripheral nerve sheath tumors, cNF have little, if any, malignant potential [1, 2, 7]. Finally, whereas pNF clearly originate within nerves and plexuses, the origin of cNF remains uncertain particularly in relation to cutaneous innervation [1, 8, 9].

The precise nature of cNF has long been debated, originally considered by many to be dysplasias, hamartomas or a dysfunctional wound-healing process [10–13]. However, over the last decade emphasis has been on cNF as “true” neoplasms [6, 14]. This is based on evidence in human cNF and pNF of a “second hit” diploinsufficient (DI) mutation of the remaining functional *NF1* allele in the SC of the lesions [15–18], with little evidence of this occurring among other cell types such as fibroblasts. These other cell types may be passively engulfed or actively recruited into the growing pNF and cNF in response to trophic factors secreted by the hyperproliferating DI nSC [19]. However, whereas mouse models with SC-specific diploid *Nf1* ablations develop pNF analogous to those seen in human NF1 patients, there had been little indication of human-like cNF in these animals [20, 21], which suggests that pNF and cNF have distinct origins.

A key clinical observation has been typically overlooked in considering the potential origin of cNF. which is NF1 patients are often afflicted with chronic itch and pain involving the skin that is independent of and even precedes the overt appearance of cNF [2, 22–25]. These sensations have largely been assumed to originate from nerve irritations caused by detected or undetected pNF, although itch has been attributed to mast cells within cNF [26–29]. However, little attention has been paid to what role, if any, that cutaneous innervation plays in the pathogenesis of cNF. Even the presence and extent of cutaneous innervation in cNF has barely been investigated [30].

To determine whether there is anything about the cutaneous innervation that was prescient to the evolution of cNF or neuropathic symptoms, we conducted an in-depth investigation of the cutaneous innervation in 3mm skin punch biopsies taken from three locations in normal appearing glabrous and hairy skin (at least 1 cm from the nearest cNF) in nineteen NF1 patients as compared to the innervation in comparable biopsies from sixteen normal volunteers. These analyses were performed with a comprehensive, integrated, multi-molecular immunofluorescence (IF) research platform referred to as the INTiDYN ChemoMorphometric Analysis (ITD-CMA) that has evolved over decades of research on normal and pathological cutaneous innervation [31–37]. ITD-CMA is an enhanced approach to the increasing use of skin biopsies to elucidate potential cutaneous mechanisms of chronic pain associated with a variety of neuropathic pain disorders [38–43].

## Materials and methods

The human research protocols were approved by the Saint Peter’s Hospital (Albany, NY) IRB for skin biopsies and by the Western IRB for collection of small cNF (s-cNF). All subjects gave written informed consent to the collection and use of the skin biopsies and cNF for research purposes. Skin biopsies, 3mm in diameter, and s-cNF were obtained by established procedures and appropriate monitoring [35]. No adverse events occurred as a result of skin biopsy and s-cNF collection procedures.

### Biopsy specimen

**Fig 1:**
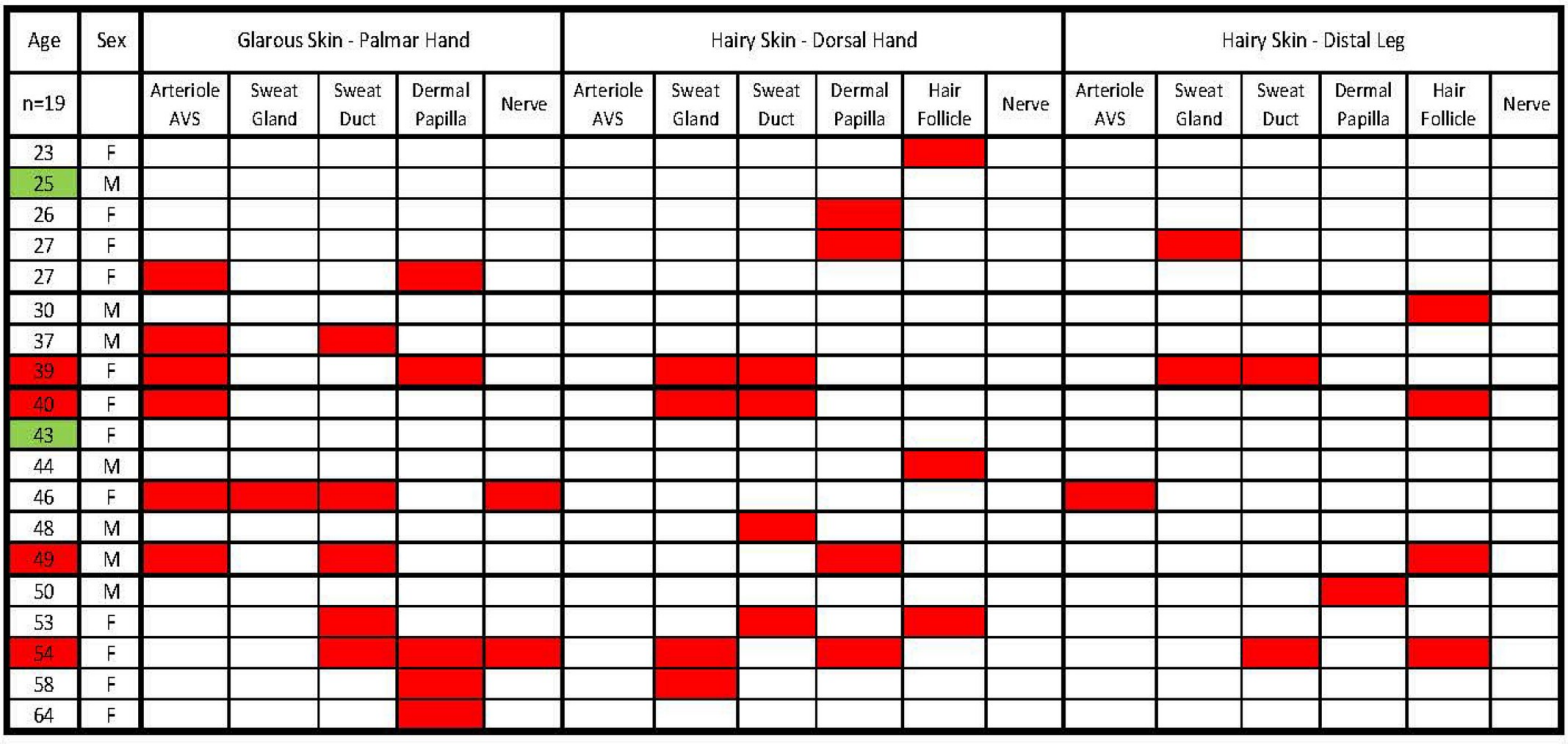
Summary of the incidence and locations of the pre-cNF among the biopsies of 19 NF1 patients. The three skin types and biopsy locations are listed in the first row. The sites where sensory endings terminate in each biopsy site are listed in the second row. Except for the first column, red boxes indicate where a pre-cNF was detected among the biopsies for each patient. In column 1, red boxes indicate those patients who had at least one pre-cNF in each biopsy. Green boxes indicate those patients who had no detectable pre-cNF.

Skin biopsies were collected from 19 NF1 patients (20–64 years old; Fig 1) and 16 genetically and somesthetically normal subjects (24–70 years old). Both groups had a similar proportion of males and females. Following clinical assessments, 3 mm skin punch biopsies were obtained under local lidocaine anesthesia from three locations of normal appearing skin (at least 1 cm from the nearest cNF) of each NF1 patient and three comparable locations of each normal subject. As assessed by ITD-CMA in prior unrelated human studies [35, 36, 39, 41], the three biopsy locations were: hypothenar palmar glabrous skin, hypothenar dorsal hairy skin, and distal leg hairy skin above the lateral malleolus. Following previously published procedures [34, 35], the biopsies were immediately immersion-fixed in 4% paraformaldehyde in 0.1M phosphate buffered saline (PBS) at pH 7.4 for 4 hours at 4°C, then rinsed and stored in PBS at 4°C. In addition, twenty s-cNF (3-6mm in diameter) surgically removed from the backs of two other female NF1 patients (32 and 35 years old) were supplied from the tissue bank maintained by the Children’s Tumor Foundation (New York, NY). These neurofibromas had been fixed overnight in 10% neutral formalin then rinsed and stored in 70% ethanol. Upon receipt at INTiDYN, the s-cNF were rinsed and stored in PBS at 4°C. Additional s-cNF from these same two NF1 patients and 11 others were flash-frozen to obtain total mRNA and sent to Sage Bionetworks (Seattle, WA) for quantitative transcriptomic functional analyses [44].

### Immunofluorescence

Following the ITD-CMA multi-label IF procedures [34, 35], the NF1 patient skin biopsies, normal subject skin biopsies, and s-cNF were cryoprotected in 30% sucrose/PBS, mounted in optimal cutting temperature media, and frozen sectioned by cryostat at a 14μm thickness. Consecutive sections were thaw-mounted, rotated sequentially across a series of at least 20 slides, such that each slide contained numerous sections from equally spaced intervals throughout the entire biopsy. The NF1 patient biopsies, normal subject biopsies, and 10 of the s-cNF were sectioned perpendicular to the skin surface. The remaining 10 s-cNF were sectioned parallel to the skin surface. All specimens were processed for integrated IF assessments using several double-label combinations of primary antibodies on alternating sections. The primary antibodies were chosen to elucidate the structural and neurochemical properties of the innervation and other components of the skin and s-cNF (Table 1). In particular, the Protein Gene Product 9.5 (PGP) antibody, which is directed against ubiquitin-C-terminal hydrolase (UCHL1), labels all cutaneous innervation. All primary antibodies were visualized by labeling with species appropriate donkey secondary antibodies conjugated with Cy3 for red fluorescence and Alexa488 for green fluorescence (Table 1) and all slides were counterstained stained with 4’,6-diamidin-2-phenylindole hydrochloride (DAPI) to reveal cell nuclei in blue fluorescence. The primary antibodies had been previously validated and used in previous studies of mouse, rat, monkeys, and humans, so controls for this study were omission of the primary antibodies on sections of the cNF, which had never been analyzed before.

**Table 1:** Chemomorphometric analysis (CMA) antibody specifications.

*Antibody*	*Species*	*Dilution*	*Source*
CGRP	rabbit	1:800	Chemicon/Millipore [ab15360]
CGRP	sheep	1:500	Abcam [ab22560]
cRET	rabbit	1:250	Abcam [ab134100]
DβH	rabbit	1:800	Chemicon/Millipore [ab145]
GAP-43	rabbit	1:1000	gift from David Schreyer
GAP-43 (monoclonal)	mouse	1:400	Abcam [ab12274]
GFRα1	rabbit	1:50	Abcam [ab8026]
GFRα1 (monoclonal)	mouse	1:200	R&D Systems [mab560]
GFRα2	rabbit	1:50	Abcam [ab8027]
GFRα2 (monoclonal)	mouse	1:200	R&D Systems [mab613]
GFRα3	rabbit	1:50	Abcam [ab8028]
GFRα3 (monoclonal)	mouse	1:200	R&D Systems [mab6701]
GFRα4 (monoclonal)	mouse	1:200	R&D Systems [mab1439]
MBP	rabbit	1:1000	Biogenesis [ab6420-2204]
NF200	rabbit	1:800	Chemicon/Millipore [ab1989]
NF200 (monoclonal)	mouse	1:400	Sigma [ab142]
NGFR (p75) (monoclonal)	mouse	1:40	Abcam [ab3125]
NPY	sheep	1:800	Chemicon/Millipore [ab1583]
NRG1	rabbit	1:100	Abcam [ab191139]
NSE (monoclonal)	mouse	1:100	Abcam [ab16808]
pChAT	rabbit	1:10,000	gift from Hiroshi Kimura
PECAM (monoclonal)	mouse	1:50	DAKO [abMO823]
PGP9.5	rabbit	1:800	Cedarlane (UltraClone Ltd) [RA95101]
PGP9.5 (monoclonal)	mouse	1:200	Cedarlane (UltraClone Ltd) [31A3]
S100Beta	rabbit	1:500	Abcam [ab52642]
S100Beta (monoclonal)	mouse	1:250	Abcam [ab11178]
SOX10	rabbit	1:200	Abcam [ab180862]
TGFβ1 (monoclonal)	mouse	1:250	Abcam [ab190503]
TrkA	sheep	1:500	Abcam [ab72029]
TrpA1	rabbit	1:400	Astra-Zeneca
TrpV1	rabbit	1:500	Abcam [ab3487]
VAChT	goat	1:1000	Santa Cruz Biotechnology [sc7717]
***Cy3 secondary abs***			***Jackson Immuno Research***
goat IgG	donkey	1:500	705-165-003
rabbit IgG	donkey	1:500	711-165-152
sheep IgG	donkey	1:500	713-165-003
mouse IgG	goat	1:500	115-165-003
***Alexa488 secondary abs***			***Life Technologies***
rabbit IgG	donkey	1:250	A21202
sheep IgG	donkey	1:250	A21206
mouse IgG	donkey	1:250	A11015
***Histochemical Reagents***		***Dilution***	***Source***
DAPI		100ng/ml	Sigma [D-9542]

All primary antibodies are polyclonal unless noted otherwise

### Digital imaging

**Fig 2:**
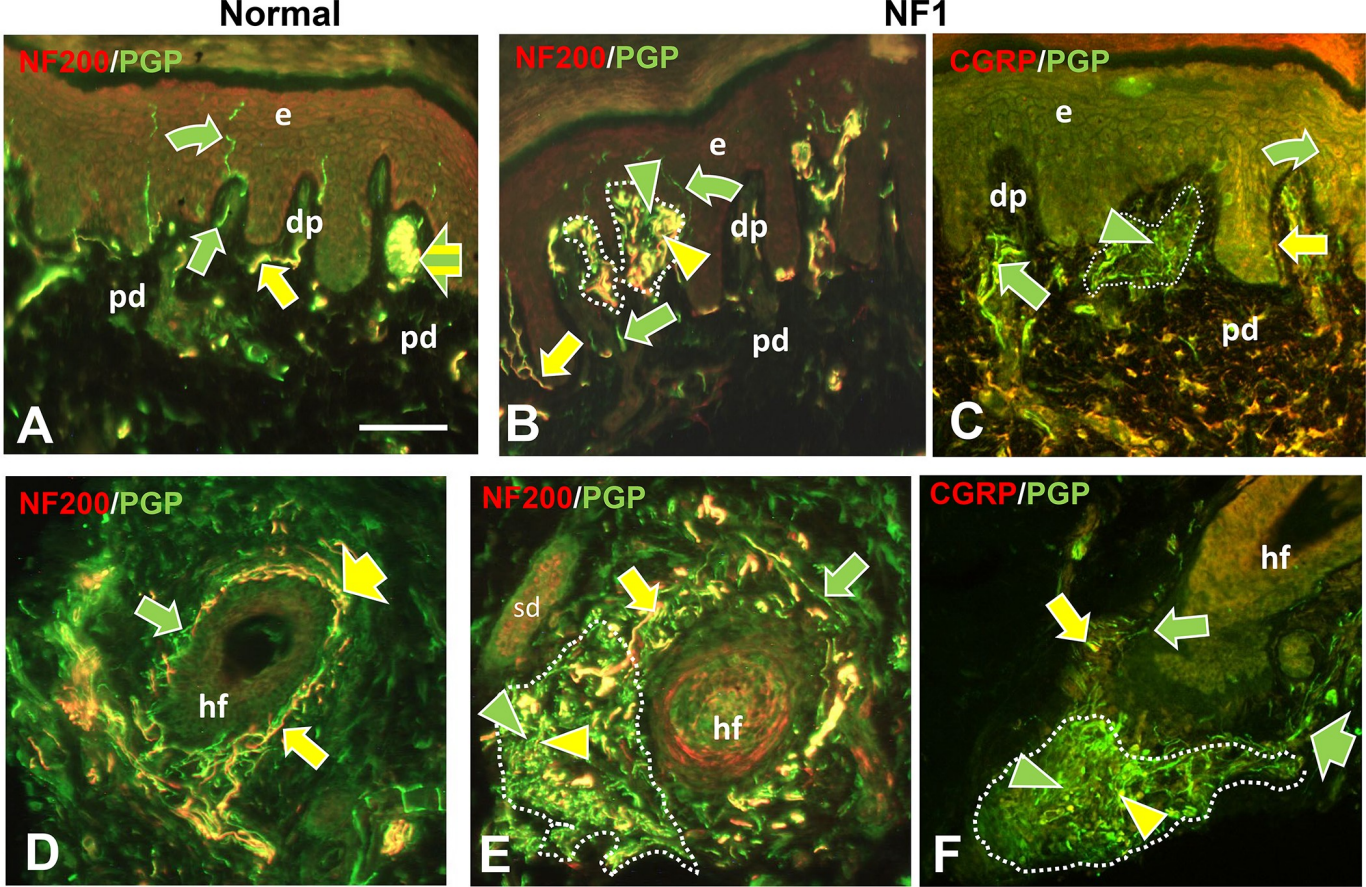
Immunofluorescence profile of pre-cNF among dermal papilla and hair follicles. Immunolabeling of NF1 patient skin biopsies revealed pre-cNF in dermal papillae (dp in B, C) that are invaginations of papillary dermis (pd) into the epidermis (e), and adjacent to hair follicles (hf in E, F). A, D: Innervation of dp and hf in normal subjects. Normally, dp and hf are innervated by a mix of large and small-caliber fibers, all of which label for PGP with the larger caliber fibers co-labeling for NF200 (A, B, D, E; yellow arrows) and a contingent of small-caliber fibers co-labeling for CGRP (C, F; yellow arrow). Other small caliber fibers only labeled with PGP (green arrows). In some cases, such as a Meissner’s corpuscle (A; broad yellow and green striped arrow), endings of large-caliber NF200-positive and small-caliber NF200-negative terminals are intermingled. Arrowheads indicate abnormal extremely fine-caliber, dense innervation within focal pre-cNF circumscribed by dotted outlines. In pre-cNF, the densely packed, extremely small-caliber fibers labeled for PGP. A high proportion co-labeled with NF200 (B, E; yellow compared to green arrowheads). Very few co-labeled for CGRP (C, F). Scale bar = 50μm.

**Fig 3:**
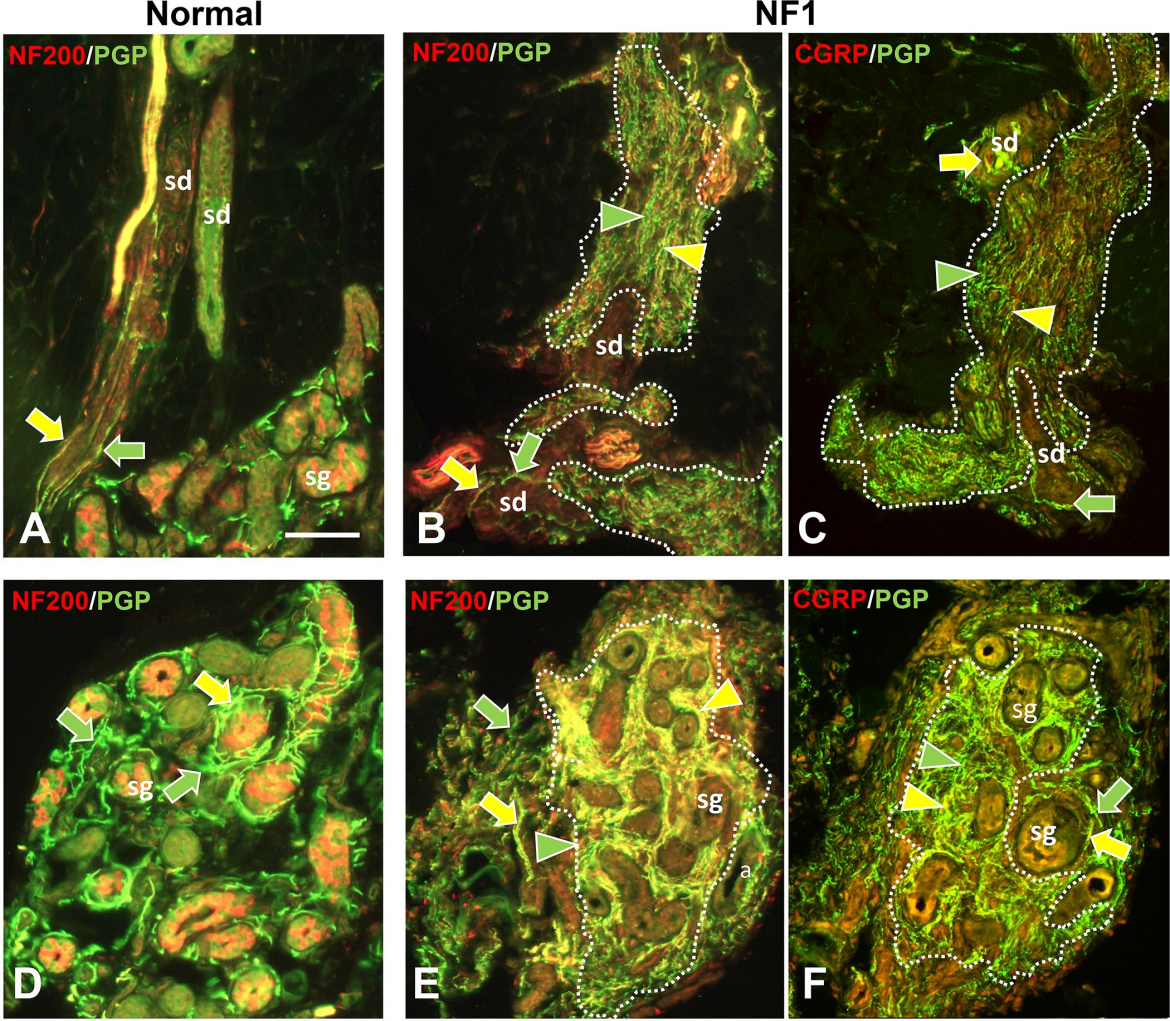
Immunofluorescence profile of pre-cNF among sweat ducts and glands. Immunolabeling of NF1 patient skin biopsies revealed pre-cNF engulfing sweat ducts (sd in B, C) and infiltrating sweat glands (sg in E, F). A, D: Innervation of sd and sg in normal subjects. The sweat ducts normally have an extremely sparse innervation with NF200-positive and NF200-negative fibers (A, B; yellow and green arrows) and virtually no fibers with CGRP (C: green arrow). Normal sweat gland tubules are each surrounded by a loose tangle of fibers of which nearly all revealed by PGP labeling (D-F; green arrows) are cholinergic sympathetic with a sparse contingent of sensory fibers that co-label for NF200 or CGRP (D-F; yellow arrows). In pre-cNF engulfing sweat ducts or embedded within sweat glands (dotted outlines), the densely packed, extremely small-caliber fibers labeled for PGP. A high proportion co-labeled with NF200 (E; yellow compared to green arrowheads). Very few co-labeled for CGRP (F; yellow arrowhead). Scale bar = 50μm.

**Fig 4:**
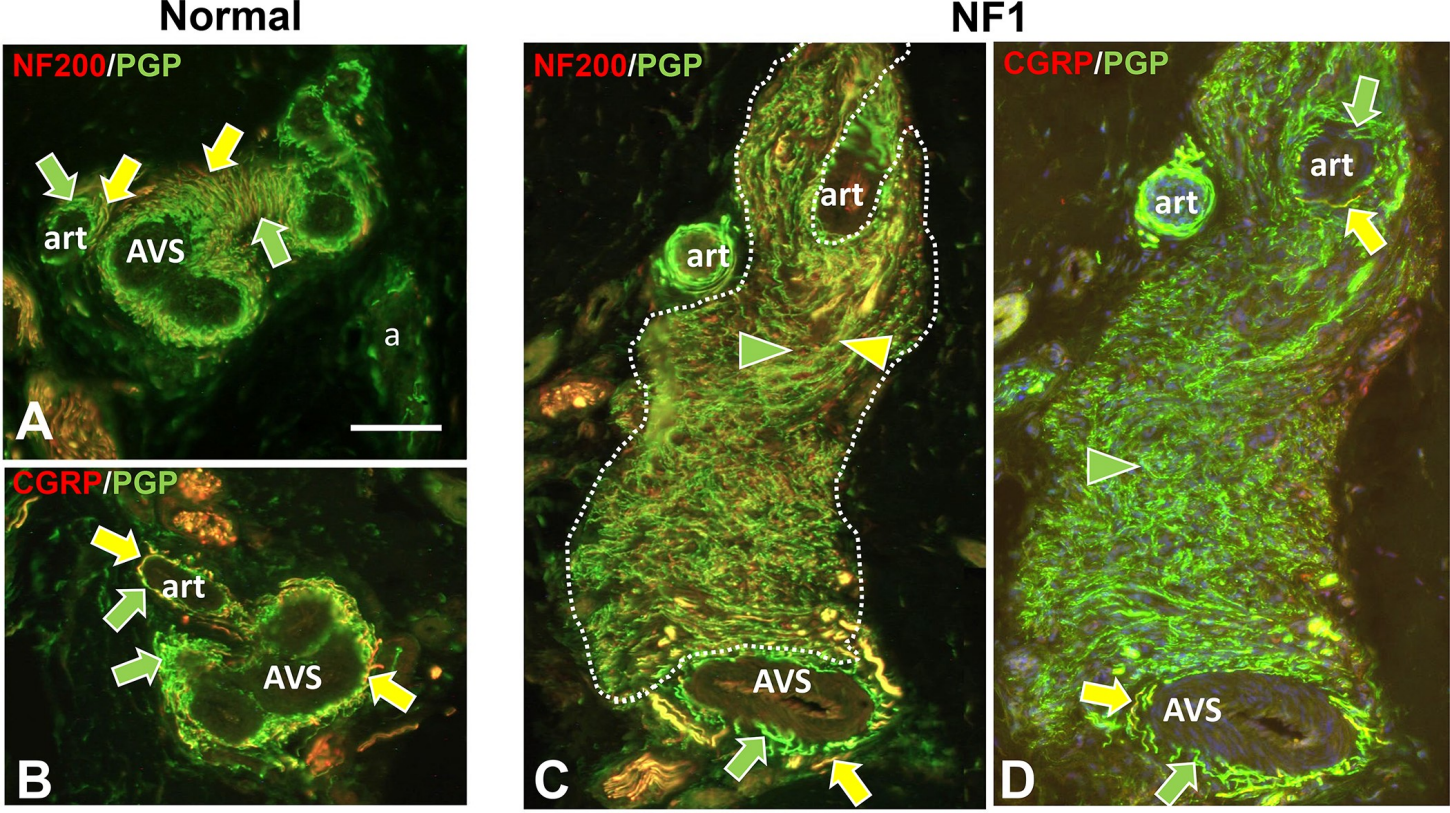
Immunofluorescence profiling of pre-cNF among arterioles and AVS. Immunolabeling of NF1 patient skin biopsies revealed pre-cNF (C, D) occurring at the border of arterioles (art) and arteriole venule shunts (AVS). A, B: Innervation of art and AVS in normal subjects. The perimeters of the arterioles and especially the AVS normally have a dense PGP-labeled, small-caliber innervation that consists of numerous sensory endings of which nearly all co-label for CGRP (B, D; yellow arrows) as well as most co-labeling for NF200 (A, C; yellow arrows). The remainder of the innervation labels only for PGP (green arrows) consists of some sensory fibers and mostly noradrenergic sympathetic fibers. In the pre-cNF (dotted outlines) located at the perimeter of art and AVS, the exceptionally small-caliber and extremely dense innervation has a unique and very high proportion that co-label with NF200 (C; yellow arrowhead), but hardly any that co-label for CGRP. Scale bar = 50μm.

**Fig 5:**
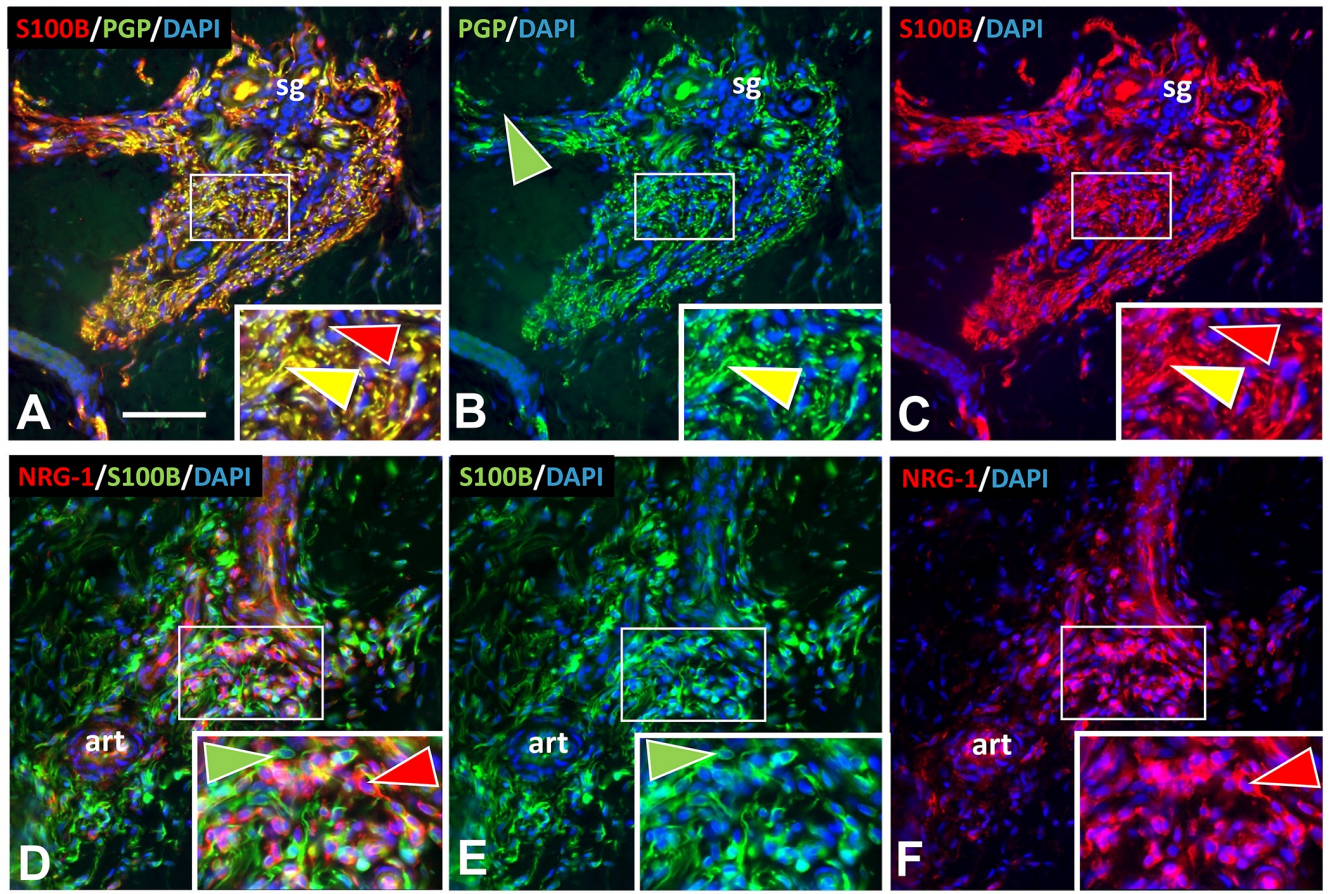
SC and other cells in pre-cNF. Immunolabeling revealed the presence in pre-cNF of abnormal concentrations of SC immunolabeled for S100B (A-C) and another non-neuronal cell type immunolabeled for NRG-1 (D-F). Inserts are 2X enlargements of sites in the small white rectangles. A-C: In this pre-cNF located at the edge of a sweat gland (sg), dense aberrant axons labeled for PGP are intimately lined with SC processes labeled with S100B (yellow arrowheads). S100B is expressed in SC bodies (red arrowheads). D-F: In this pre-cNF located at the edge of a small arteriole (art), S100B-labeled SC bodies and their processes (green arrowheads) are completely distinct from clusters of other cells with few processes that label for NRG-1 (red arrowheads). Scale bar = 50μm.

**Fig 6:**
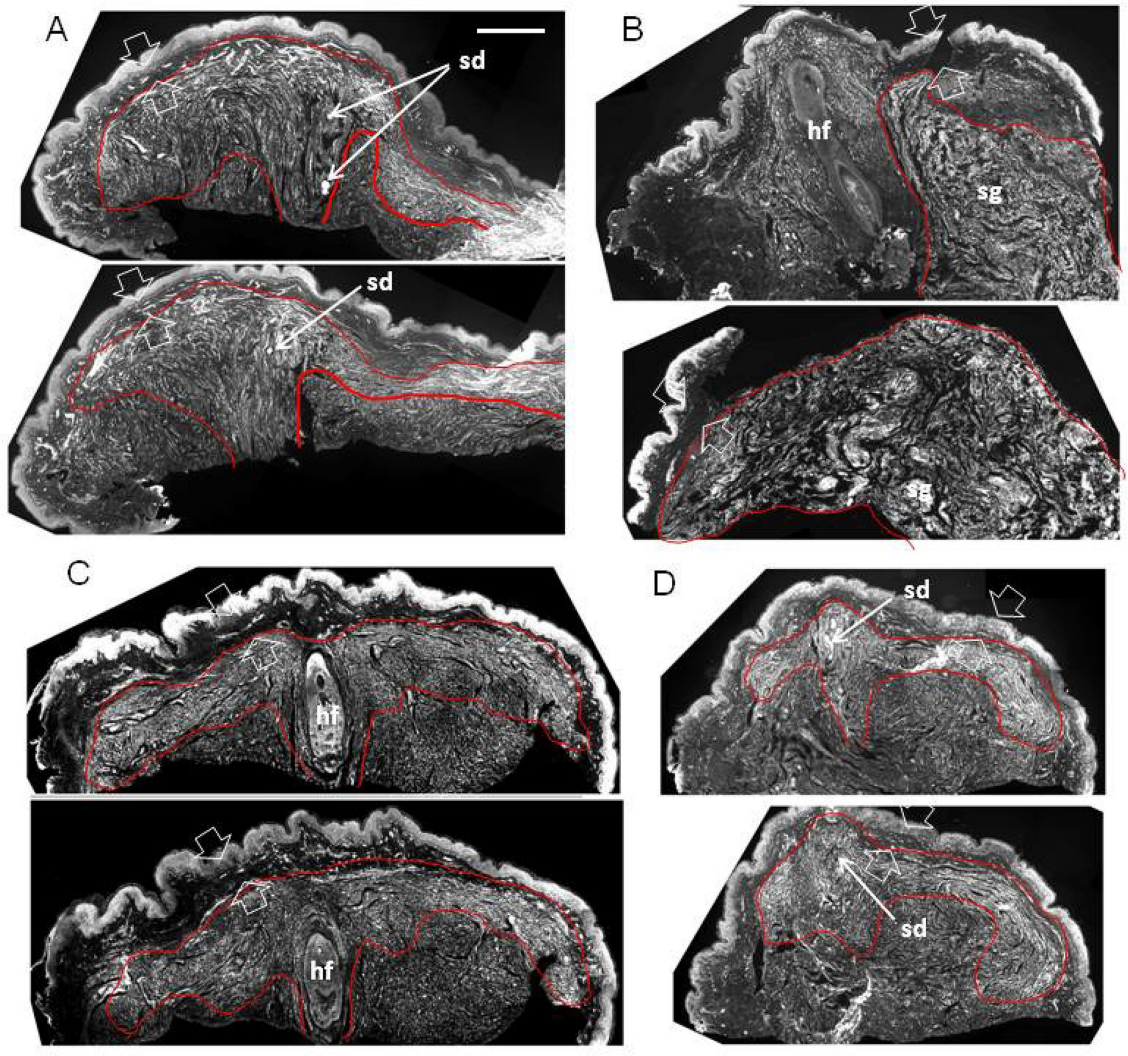
Structural organization of s-cNF around an adnexal core (perpendicular). Immunolabeling reveals the adnexal core of four s-cNF as seen in each of two of the serial sections each cut perpendicular to the s-cNF surface and immunolabeled for PGP. Concentrated areas of aberrant dense innervation are outlined in red and typically consist of fibers ascending adjacent to the adnexal core structure and spreading out under and parallel to, but not penetrating the papillary dermis (between broad arrows). In A and D, the core structure is a sweat duct; in C, a hair follicle. In B, a hair follicle is pushed to the side by excessive nerve fibers originating from a deeper sweat gland. Scale bar = 500μm.

**Fig 7:**
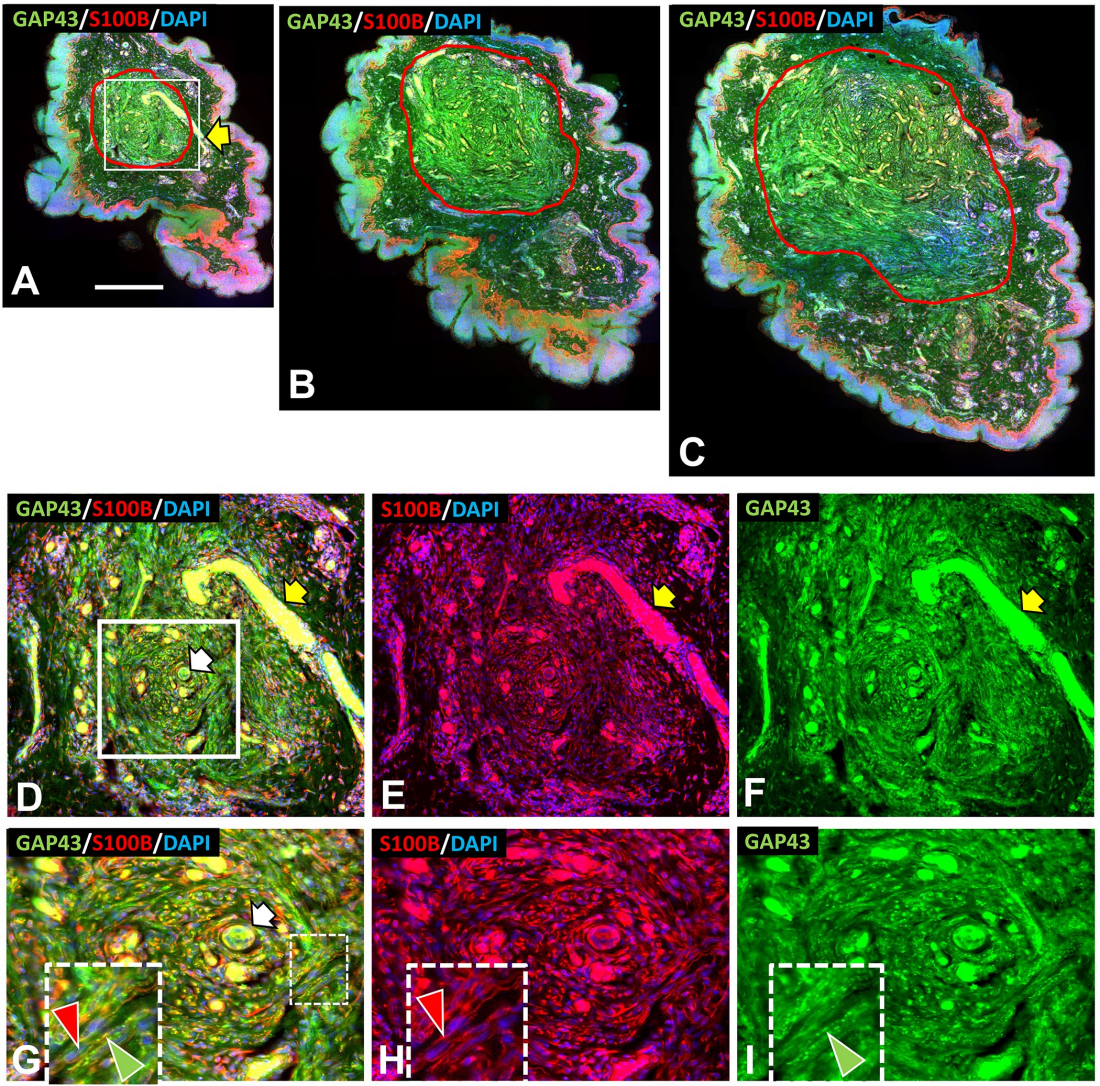
Structural organization of an s-cNF around an adnexal core (parallel). Immunolabeling reveals the adnexal core of a 4mm s-cNF sectioned parallel to the epidermal surface and co-labeled for GAP-43 (green), S100B (red), and DAPI (blue). Small blood vessels filled with albumen autofluorescence (yellow arrow) A-C: Complete montages of 3 sections at successively deeper levels. The red contours outline the perimeter of a dense concentration of small-caliber fibers oriented circumferentially around a core. The area in the white square of A is shown at 2X magnification in D-F. D-F: Increased magnification reveals a sweat duct (white arrow) surrounded by small-caliber fibers cut in cross-section at the core. The fibers shift to a circumferential orientation around this core. S100B-labeled SC (E) intimately match the orientations and concentrations of the fibers labeled for GAP-43. The area in the white square of D is shown at still a 2X higher magnification in G-I. G-I: An increased magnification of the sweat duct and associated small-caliber fibers cut in cross-section at the core of the s-cNF. Insets in G-H are a further 2X magnification of the broken line area in G that show the intimate association of SC and their processes (red arrowheads) with the aberrant small-caliber fibers (green arrowheads). Scale bar = 500μm.

**Fig 8:**
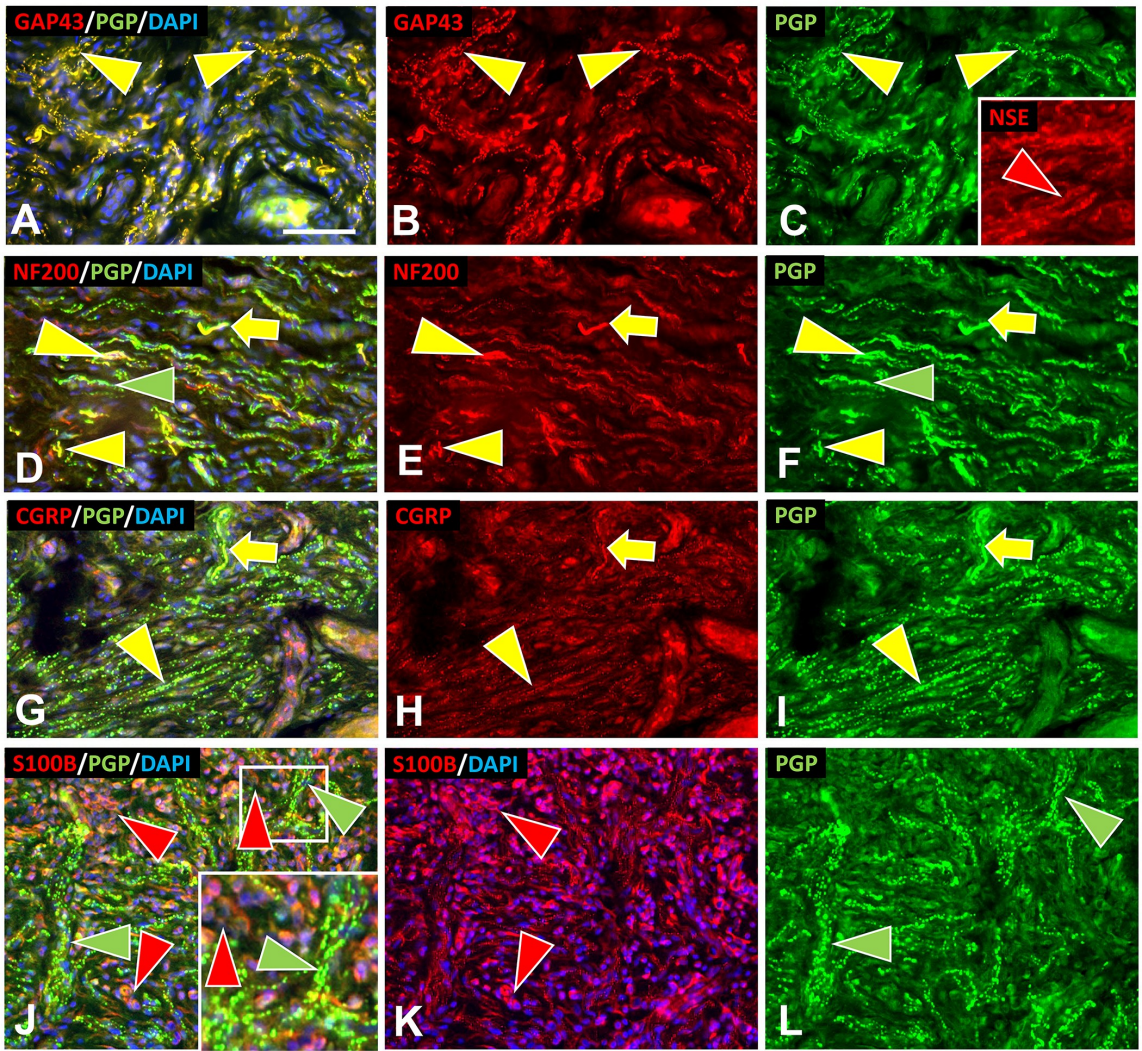
Multi-molecular immunofluorescence characteristics of s-cNF aberrant innervation. Multi-molecular immunolableing double-labeled with combinations for PGP together with either GAP-43, NSE, S100B, NF200, or CGRP revealed aberrations among s-cNF. A-C: Virtually all of the aberrant innervation co-labels for PGP and GAP-43 (yellow arrowhead). NSE is more widely expressed likely on innervation and SC (red arrowhead). D-F. A high proportion of the aberrant fibers co-label for NF200 (yellow arrowheads). Others only label for PGP (green arrowheads). Yellow arrows indicate what normal NF200 innervation should look like. G-I: Yellow arrows indicate fibers that are typical of normal CGRP and PGP co-labeling. Most of the aberrant fibers have faint tiny punctate labeling for CGRP (yellow arrowheads). Yellow arrows indicate what normal CGRP innervation should look like. J-L: Aberrant concentrations of PGP-labeled fibers (green arrowheads) are intermingled with SC (red arrowheads). Inset is a 2X of the small square. Scale bar = 25μm.

High resolution epifluorescence images were captured utilizing an Olympus BX51 microscope equipped with a Hamamatsu ER camera or an Olympus Optical Provis AX70 microscope equipped with a Hamamatsu C11440 camera (e.g., Figs 2–8). Each microscope system was equipped with conventional fluorescence filter cubes for specific excitation and emission spectra required for blue/green/red fluorophore channel separation, a linear focus encoder, and a 3-axis motorized stage, interfaced with Neurolucida software (MBF Bioscience, Essex, VT), enabling seamless high resolution whole section montages and complete off-line or real-time image mapping/measuring of selected elements. Image montages (e.g., Figs 6 and 7) were collected using identical camera settings for each label across the entire specimen cohort. Co-labeling for various antigens was assessed using the application Photoshop (Adobe Systems, San Jose, CA) to analyze each color channel. For these studies, the analysis consisted of visual interrogation of the biopsies to examine the morphological and immunocytochemical characteristics of s-cNF innervation and intrinsic cellular components. In all figures showing fluorescent immunolabeling, the antigens for the primary antibodies are indicated in the upper left corner in the color of the corresponding secondary antibody. All primary antibodies were validated in prior studies among the various co-authors. Control sections from skin biopsies and s-cNF were processed only with secondary or no antibodies to assess for nonspecific secondary antibody immunolabeling or autofluorescence (Section A in S1 Text and S1 Fig).

### Gene expression analysis

**Fig 9:**
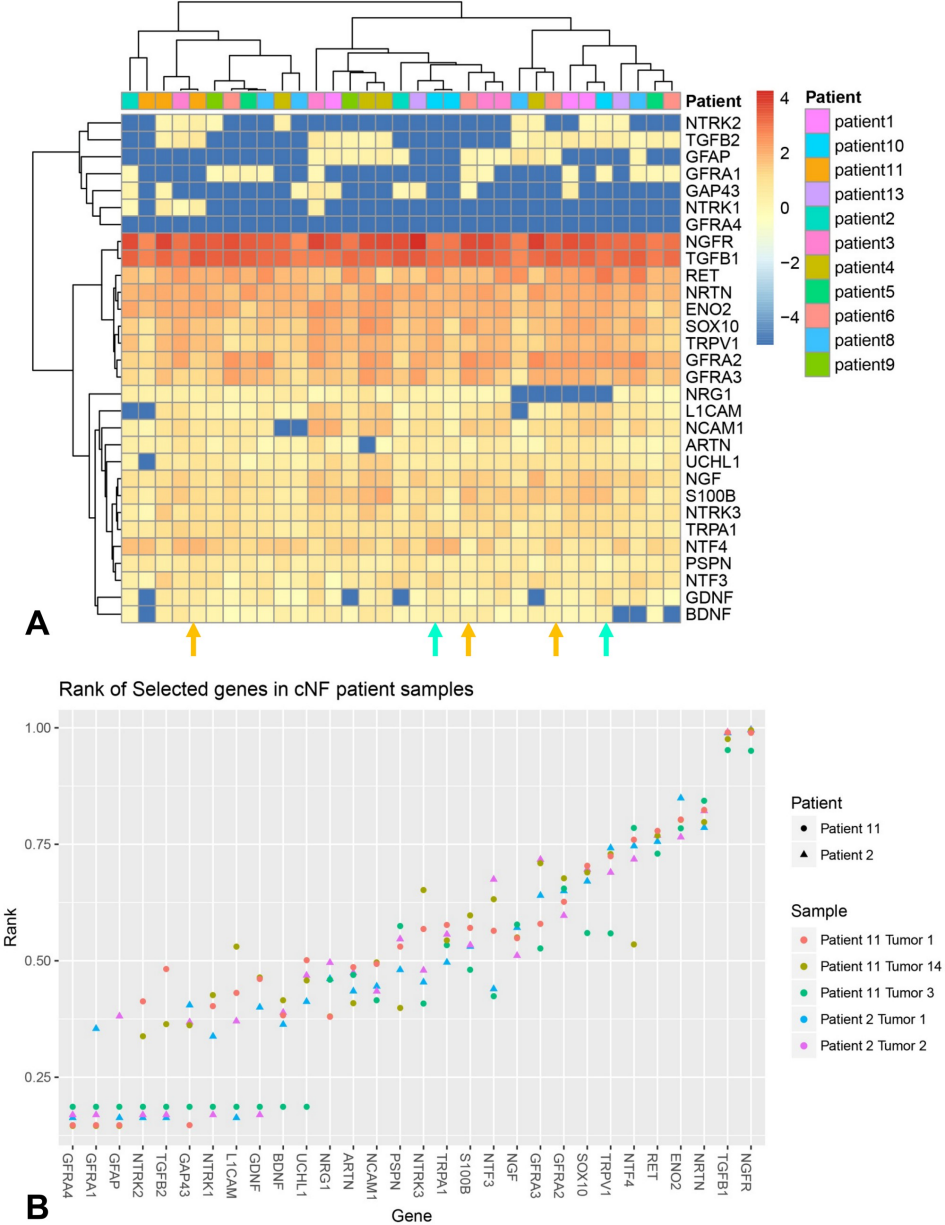
mRNA expression for most of immunochemically assessed proteins in this study. A: Shows the absolute levels in multiple s-cNF from 13 different patients color coded by Z-score, with blue representing poorly expressed genes and red representing highly expressed genes in each biopsy from each patient. Patient key is on the right. The s-cNF biopsies for the ITD-CMA are from Patient 2 (turquoise arrows) and Patient 11 (orange arrows). B: Shows the relative mRNA expression levels for only two s-cNF from Patient 2 and three s-cNF from Patient 11.

Independent from and blinded to the ITD-CMA results, gene expression measurements of select target genes were captured in up to four flash frozen s- cNF from each of the two NF1 patients that were the source of the s-cNF for the ITD-CMA analyses, as well as from each of 11 additional NF1 patients [44]. Specifically, normalized counts were visualized in a heatmap representation using the R pheatmap function and library. To compare the relative expression of specified genes of interest in the s-cNF that are of interest based on the ITD-CMA results, a plot of the relative rank of each gene (x-axis) was created for each patient sample. The higher the rank, the more abundant the transcript was in that sample (Fig 9).

**Fig 10:**
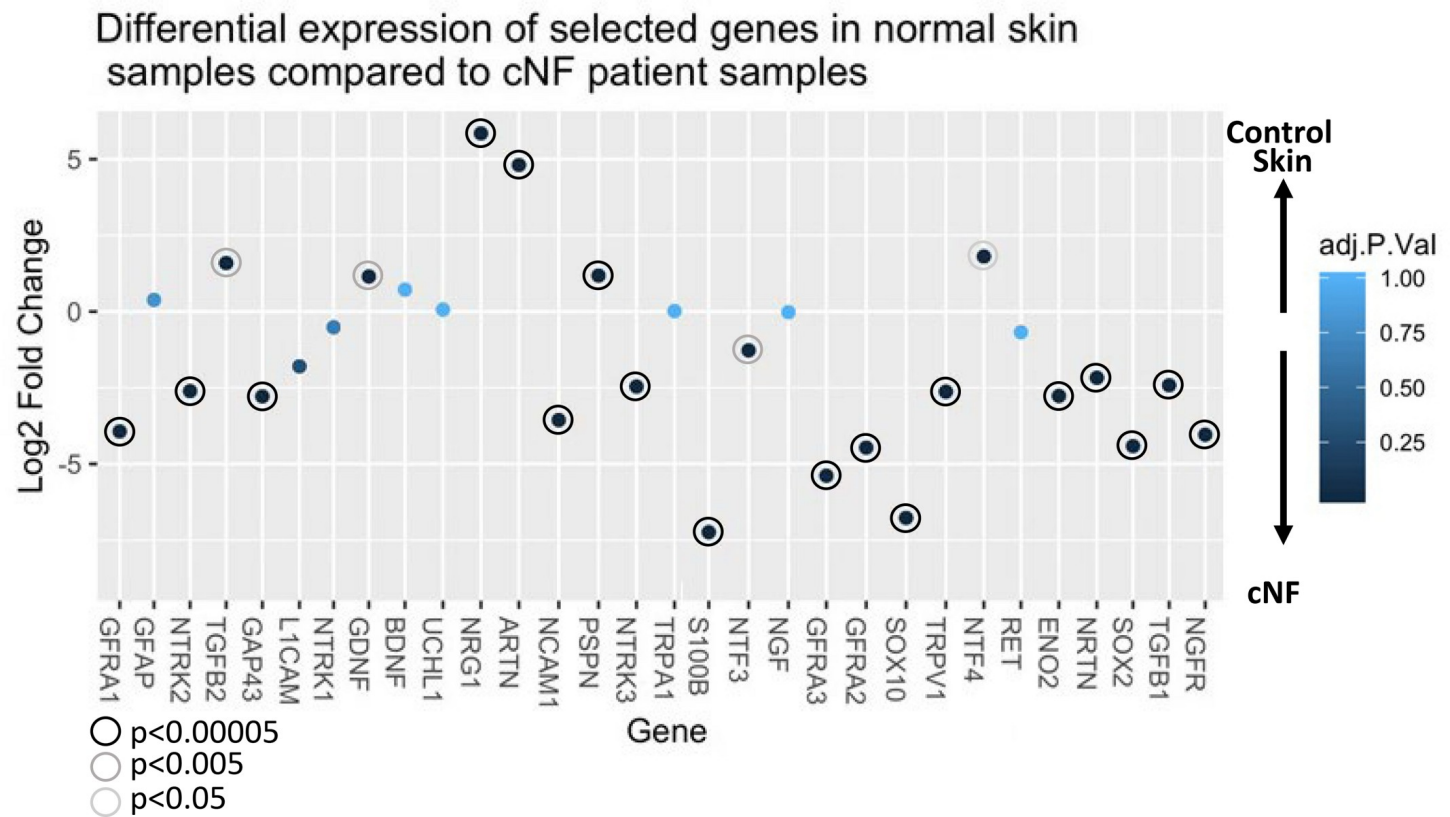
Differential expression values of selected genes. Comparisons were performed to determine differential expression values of selected genes in Fig 9 compared to 33 normal skin controls. Negative values indicate up-regulation in s-cNF while positive values confer down-regulation. Corrected P-value is indicated by color of the point. Significantly different data points are encircled.

Published transcript levels were also compared to 33 normal skin controls derived from the ENCODE consortium using the LIMMA tool to identify genes that were differentially expressed between the cNF patient samples and the skin controls [45]. In total, 6996 genes out of 28,517 transcripts were differentially expressed (adj. p<0.01), suggesting a possible batch effect. However, differential expression values of those genes of particular interest based on ITD-CMA results are depicted in Fig 10. Genes that exhibit a negative log2 fold change are up-regulated in cNFs compared to normal skin.

### Mouse model

The *DhhCre;Nf1*^*fl/fl*^ model was described previously [21]. All animal procedures were approved by the Animal Care and Use Committee of Cincinnati Children’s Hospital Medical Center. Mice were housed in temperature- and humidity-controlled facilities with 12-hour dark-light cycles and free access to food and water. Mice were largely on the C57Bl/6 strain background and were genotyped by PCR [46, 47]. Briefly, male *DhhCre* mice were bred onto the female *Nf1*^*fl/fl*^ background to obtain the F1 generation (*DhhCre;Nf1*^*fl/+*^). Female *Nf1*^*fl/fl*^ mice were then bred with male *DhhCre;Nf1*^*fl/+*^ mice to obtain *DhhCre;Nf1*^*fl/fl*^ mice. *DhhCre;Nf1*^*fl/+*^ littermates were used as controls. Analyzed at 7–9 months old, *DhhCre;Nf1fl/fl* mice develop plexiform neurofibromas, and enlargements in cutaneous nerves described as Grade 1 neurofibroma with numerous spindle shaped S100+ cells, abundant collagen, and mast cells. Hindpaws from 4 of these *DhhCre;Nf1*^*fl/fl*^ mice, that had been perfusion fixed with 4% PFA, were amputated at the ankle. The hairy dorsal skin and glabrous plantar skin was removed and was sectioned and processed for ITD-CMA as described above for the human skin biopsies.

## Results

### Detection and incidence of presumptive cNF precursors (pre-cNF)

In at least one biopsy from 17 of the 19 NF1 patients (Fig 1), immunolabeling for PGP9.5 revealed one or more micro-lesions containing small, discreet dense clusters of small-caliber nerve fibers (Figs 2–4, dotted outlines). The clustered nerve fibers immunolabeled for the axon growth marker GAP-43, consistent with axon sprouting (see below). Eventually interpreted as precursors to cNF (pre-cNF), these neural micro-lesions were not observed in any of the normal subject biopsies and had not been observed previously in other studies of pathological human skin biopsies [34, 35, 39, 41, 48]. Four patients had at least one micro-lesion in all three biopsies.

Nearly all of these micro-lesions were intimately associated with adnexal skin structures that are sites where various combinations of cutaneous innervation normally terminate [34, 36, 37, 49]. These adnexae included dermal papillae that invaginate into the epidermis (Fig 2A–2C), hair follicles (Fig 2D–2F), sweat ducts (Fig 3A–3C), sweat glands (Fig 3D–3F), and arterioles and arteriole-venule shunts (AVS) (Fig 4). A total of 45 microlesions were detected among all the biopsies from NF1 patients, with some biopsies containing more than one microlesion, each involving its own adnexa (Fig 1).

In each micro-lesion, S100B immunolabeling revealed numerous presumptive nSC intimately affiliated with the clustered innervation (Fig 5). Given that cNF are known to consist overwhelmingly of nSC, this suggested that the micro-lesions might be pre-cNF. Therefore, we hypothesized that an adnexal structure would be present within overt s-cNF, accompanied by excessive innervation and nSC.

### Structural organization of s-cNF

Consistent with the pre-cNF hypothesis, immunolabeling of serial sections from numerous 3–6 mm cNF, cut perpendicular or parallel to the skin surface (Figs 6 and 7, respectively), revealed that each had an adnexal structure imbedded at the core that was associated with a massive dense concentration of aberrant innervation that extensively co-labeled for GAP-43 and PGP (Figs 7A–7C and 8A–8C). Numerous S100B-positive presumptive nSC were densely intermingled with the aberrant innervation (red and green arrowheads in Figs 7 and 8J–8L, respectively). The aberrant innervation ascended along or within the adnexal core structure and then expanded horizontally and circumferentially across the full width of the s-cNF. This organization was especially obvious in perpendicular sections demonstrating hair follicles and sweat ducts (Fig 6A, 6C and 6D). The lateral expansion was restricted to the loose reticular dermis, as though blocked and deflected from invading the thin, compact layer of papillary dermis. In some cases, a hair follicle was clearly the adnexal core (Fig 6C), but in other cases a hair follicle was entrapped by innervation originating and spreading from another adnexal structure, such as a deep sweat gland (Fig 6B).

### Multi-molecular characterization of pre-cNF and s-cNF innervation

#### Normal innervation

The next objective was an in depth ITD-CMA characterization of the aberrant pre-cNF and s-cNF innervation using certain antibody combinations to identify different functional types of innervation, as documented in previous publications [31, 33–37, 49, 50]. Arrows in Figs 2–4 indicate how normal combinations of innervation appear in biopsy sections after immunolabeling. The known types of innervation terminating at the cutaneous adnexal target structures in normal skin include: 1) various types and combinations of sensory endings supplied by thin-caliber, unmyelinated axons (C fibers); 2) slightly larger caliber, thinly myelinated axons (Aδ fibers); and 3) much larger caliber, thickly myelinated axons (Aβ fibers). Subsets of C and Aδ fibers are implicated in pain mechanisms and are referred to as nociceptors [49]. Additionally, sweat glands are also innervated mostly by thin-caliber, unmyelinated cholinergic sympathetic fibers, while arterioles and AVS are also innervated by thin-caliber, unmyelinated noradrenergic sympathetic fibers.

As documented previously for skin biopsies from normal subjects [34, 36, 37], all types of innervation immunolabeled for PGP9.5. Presumptive thin-caliber Aδ and large-caliber Aβ fibers co-labeled for 200 kD neurofilament protein (NF200; yellow arrows, Figs 2A, 2B and 2D–2F; 3A, 3B, 3D and 3E; 4A and 4C) and Myelin Basic Protein (MBP; not shown, but see S3D–S3F Fig). Presumptive C fibers lacked NF200 immunolabeling, and consisted of two major subtypes: 1) “peptidergic”, that co-labeled for CGRP, and 2) “nonpeptidergic” that lacked CGRP (yellow and green arrows, respectively; Figs 2C; 3C and 3F; 4B and 4D). As documented previously [36], sweat glands were densely innervated by small-caliber fibers (green and yellow arrows, Fig 2D) of which the vast majority were unmyelinated cholinergic sympathetic fibers that labeled for pChAT and VAChT (not shown). A small contingent consists of peptidergic and nonpeptidergic C fibers of which some immunolabeled for NF200 (yellow arrows, Fig 2D–2F). Arterioles and AVS also have a dense small-caliber innervation (green and yellow arrows, Fig 4A and 4B) which was mostly noradrenergic sympathetic fibers that co-labeled for DβH and NPY (not shown) [35, 36]. A large proportion was sensory consisting mostly of peptidergic C and Aδ fibers and a smaller proportion of nonpeptidergic C fibers (yellow arrows, Fig 4A and 4B).

As documented previously, virtually all thin-caliber sensory and sympathetic fibers normally terminate as well-ordered simple, branched or unbranched free nerve endings (FNE), while Aβ fibers terminate as site specific, morphologically unique specialized endings [34, 37, 49, 51]. For example, certain small-caliber C fibers terminated among epidermal keratinocytes as intraepidermal nerve fibers (IENF) (curved arrows, Fig 2A–2C). A mix of Aβ and C fibers terminated as Meissner corpuscles in dermal papillae (broad arrow, Fig 2A) [52] and as tooth-like lanceolate endings around the perimeter of hair follicles (broad arrows, Fig 2D and 2F) [31, 34, 49].

#### Aberrant pre-cNF innervation

Double-label combinations revealed that the aberrant dense innervation in pre-cNF (arrowheads, Figs 2–4), immunolabeled for PGP, had an especially thinner caliber than any small-caliber innervation in biopsies from normal subjects and was negative for MBP, negative for cholinergic, and negative for noradrenergic properties. Relatively little of the aberrant innervation co-labeled for CGRP, indicating that the vast majority of pre-cNF innervation is composed of nonpeptidergic C fibers and their FNE (green arrowheads). Importantly, despite the lack of MBP labeling, a substantial proportion of the innervation did label for NF200, which is not normally expressed in mature unmyelinated C fibers [34, 37, 53]. One possibility for NF200 labeling in pre-cNF may be the presence of unmyelinated branches sprouting from Aδ or Aβ fibers (Section B in S1 Text). Alternatively, NF200 is normally upregulated in all types of innervation during development before myelination occurs, but normally only persists in mature Aδ and Aβ fibers that become myelinated [53–55]. NF200 also upregulates among all fiber types including C fibers during regeneration [56].

Virtually all of the aberrant pre-cNF innervation also co-labeled with GAP-43 in alternating sections (not shown, but see Fig 7A–7C), which is expressed during normal axon growth, plasticity, regeneration, and pathological sprouting [34, 37, 57]. Like NF200, GAP-43 is expressed in all fiber types during normal development, but unlike NF200, GAP-43 normally downregulates in Aδ and Aβ fibers as they mature and stabilize [37, 58]. Unlike in mature myelinated fibers, GAP-43 continues expression in most mature unmyelinated fibers–sympathetic and sensory–indicating normal constant remodeling [34, 37, 59, 60]. Therefore, the NF200 expression among the aberrant, extremely fine caliber fibers of the pre-cNF could be indicative of an excessive sprouting of nonpeptidergic C fibers and/or *de novo* unmyelinated sprouting of destabilized Aδ or Aβ fibers.

#### Aberrant s-cNF innervation

As with pre-cNF, virtually all of the innervation in s-cNF was small caliber and co-labeled for PGP and GAP-43 (Figs 7 and 8A–8C). A substantial proportion of the PGP-labeled fibers co-labeled for NF200 (Fig 8D–8F), but lacked MBP (not shown). CGRP was barely detectable among aberrant fibers as compared to normal peptidergic C fibers (Fig 8G–8I). Among the aberrant innervation in s-cNF affiliated with a sweat gland core, very little labeling was found for the cholinergic markers, pChAT and VAChT (not shown). Likewise, little of the aberrant innervation in s-cNF affiliated with arterioles or AVS expressed the noradrenergic markers, DβH or NPY (not shown). Therefore, as observed in the pre-cNF, the vast proportion of the aberrant innervation in s-cNF appears to be highly sprouted nonpeptidergic C fibers, perhaps with some unmyelinated branches of Aδ or Aβ fibers.

Given that PGP and GAP-43 are presumably produced in the sensory neurons in dorsal root ganglia (DRG), and transported anterogradely into the axons and their terminals, mRNA transcripts for *GAP-43* were virtually absent in s-cNF although moderate levels for *UCHL1* were detected (Fig 9) likely related to observations that low levels of PGP immunolabeling can be detected on nSC [61, 62]. Compared to normal skin, relative levels of transcripts in s-cNF were similar for *UCHL1* but significantly higher for *GAP-43* (Fig 10). The mRNA analyses of s-cNF revealed a moderate to high levels for *ENO2* that was significantly higher than in normal skin (Figs 9 and 10). *ENO2* translates to a pan-neuronal enzyme neuron-specific enolase (NSE). However, subsequent s-cNF immunolabeling for NSE revealed a high level expression not only on the aberrant innervation but also the excessive associated nSC (Fig 8C, inset) as reported previously [63].

### Profiling nSC and other non-neuronal cells in pre-cNF and s-cNF

#### nSC immunolabeling

Labeling for the generic SC marker S100B [31, 33, 64], revealed that virtually all of the aberrant C fibers in pre-cNF and s-cNF were closely affiliated with nSC, and that clusters of nSC could also be present in excess of associated fibers in s-cNF (Figs 5A–5C and 8J–8L). This suggests that in pre-cNF, initial nSC proliferation parallels the spouting of the aberrant innervation, whereas in s-cNF the nSC begin autonomous clonal proliferation that loses contact with the sprouting innervation.

#### SC precursor cells

**Fig 11:**
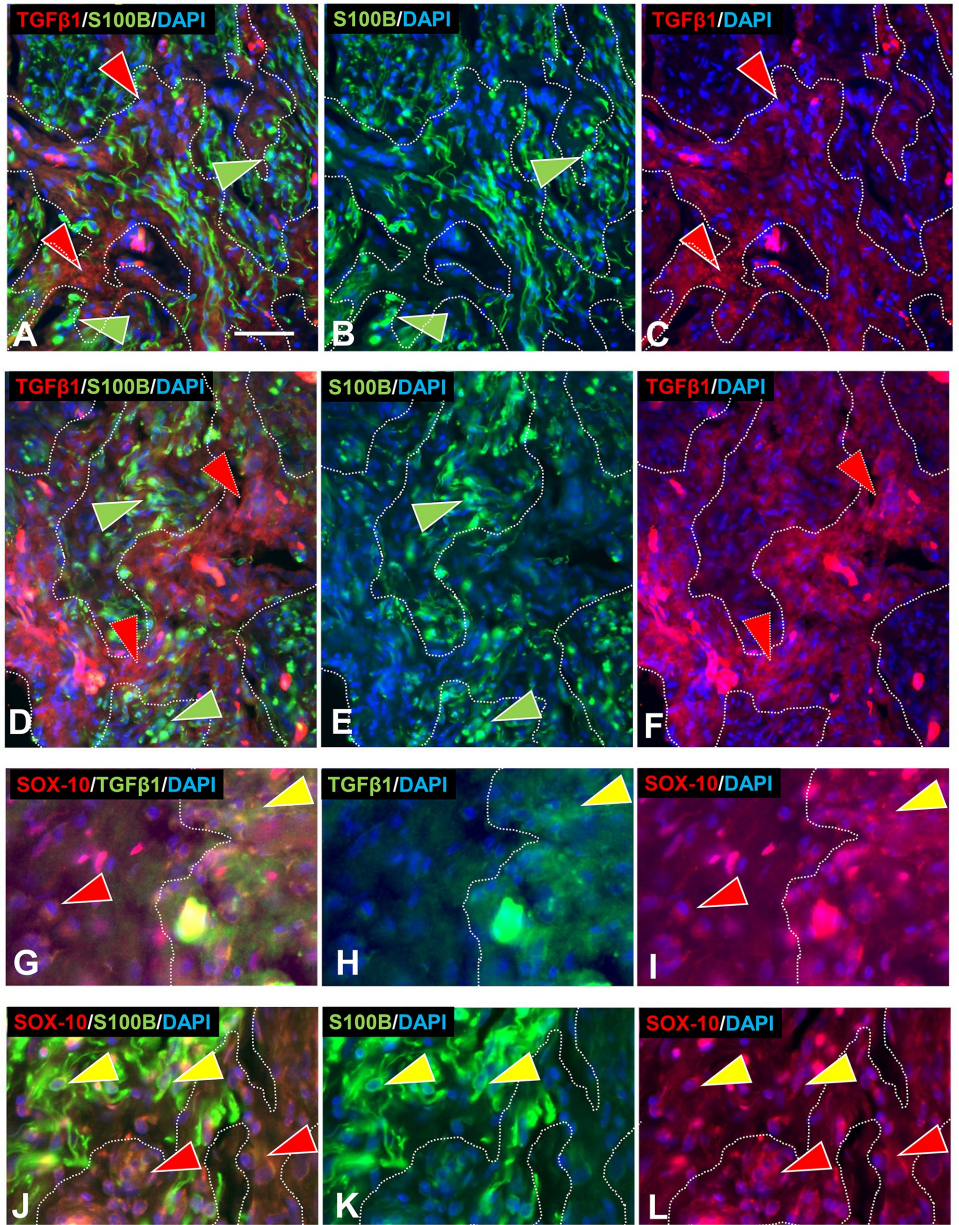
Partitioning of s-cNF into zones. Multi-molecular immunolabeling revealed a partitioning of s-cNF into zones (broken lines) of aberrant innervation and affiliated SC interdigitated with aberrant intercalated zones of densely packed clusters of another cell type that had cytoplasmic labeling for TGFβ1 (red arrowheads). Immunofluorescence double labeling for TGFβ1 (red) and S100B (green) revealed that pre-cNF (A-C) and s-cNF both have partitioning of SC immunolabeled for S100B. G-L. The SOX10 immunolabeling is co-express on TGFβ1-positive/S100 cells as well as likely definitive S100B labeled SC in pre-cNF (G-I, yellow and red arrowheads respectively) and in s-cNF (J-L, red and yellow arrowheads respectively). Scale bar = 25μm.

Transforming growth factor-beta1 (TGFβ1) has been implicated in promoting SC proliferation and differentiation especially under tumorigenic conditions [65–68]. Therefore, both pre-cNF and s-cNF were immunolabeled for TGFβ1 (Fig 11A–11F). In the pre-cNF, TGFβ1 labeling was observed on focal, densely-packed small cell clusters that did not label for S100B, and were distinct from S100B-labeled presumptive nSC (Fig 11A–11C). In s-cNF, large zones of similar densely packed small TGFβ1-positive, S100B-negative cells were intercalated with, and segregated from zones containing the aberrant innervation and affiliated S100B-positive presumptive nSC (Fig 11D–11F). In pre-cNF and s-cNF, the TGFβ1-positive/S100B-negative cells as well as the S100B-positive/ TGFβ1-negative nSC co-labeled for the transcription factor SOX-10 (Fig 11G–11L), an SC developmental lineage marker [69–72], indicating that the TGFβ1-positive/S100B-negative cells were likely proliferating SC precursors [6].

#### SC related mRNA detection

The immunolabeling results in s-cNF were supported by high levels of mRNA transcripts for *TGFB1* and *SOX10* in s-cNF from the same and additional NF1 patients, as well as moderate levels for *NCAM1* and *L1CAM* which are other, albeit less specific markers for SC progenitors (Fig 10). Relative levels of all four transcripts were expressed at especially significant higher levels in the s-cNF as compared to normal skin (Fig 10). Given the numerous S100B immunolabeled nSC in s-cNF, transcripts for *S100B* were relatively moderate but were especially significant higher levels than in control skin (Figs 9 and 10).

#### Unidentified cells

Given the affiliation between the excessive nSC and aberrantly sprouting innervation, pre-cNF and s-cNF were immunolabeled for neuregulin-1 (NRG-1), a key mitogen implicated in SC proliferation and axon/SC sensory signaling [73, 74]. Pre-cNF contained clusters of small cells that labeled with NRG-1, but not S100B, and were intermingled with the S100B labeled SC (Fig 5D–5F). The clustered NRG-1-positive cells in pre-cNF had few processes, did not resemble fibroblasts, and they did not intimately associate with the aberrant sensory fibers. By contrast, NRG-1 labeling in s-cNF only showed few similar cells which were not as densely packed and did not have the same morphology of those in the pre-cNF that labeled for TGFβ1 (not shown).

#### Mast cells, compartmentalizing cells, and sensory corpuscles

See Section C and Section D in S1 Text and S2 Fig and S3 Fig.

### Potential neural signaling mechanisms between aberrant innervation and nSC

That the aberrant innervation in pre-cNF and s-cNF was overwhelmingly composed of nonpeptidergic C fibers suggested the possible presence of ligands and receptors for two major neurotrophic factor systems that promote outgrowth of C fibers: the nerve growth factor (NGF) family and glial-derived neurotrophic factor (GDNF) family [75–78], (Section E in S1 Text). Of particular relevance to C-fibers, peptidergic C-fibers are dependent upon NGF signaling through the presence of the high affinity TrkA kinase receptor throughout development and continued adult maintenance. By contrast nonpeptidergic C fibers are initially dependent upon NGF/TrkA signaling, then switch to express and depend upon c-Ret [78–81], which is the kinase receptor common to each member of the GDNF family (GDNF, neurturin [NTRN], artemin [ARTN], and Persiphin [PSPN]) that selectively signal through c-Ret via specific co-receptors GFRα1–4, respectively [52, 78–80, 82, 83].

#### GDNF family of neurotrophins

**Fig 12:**
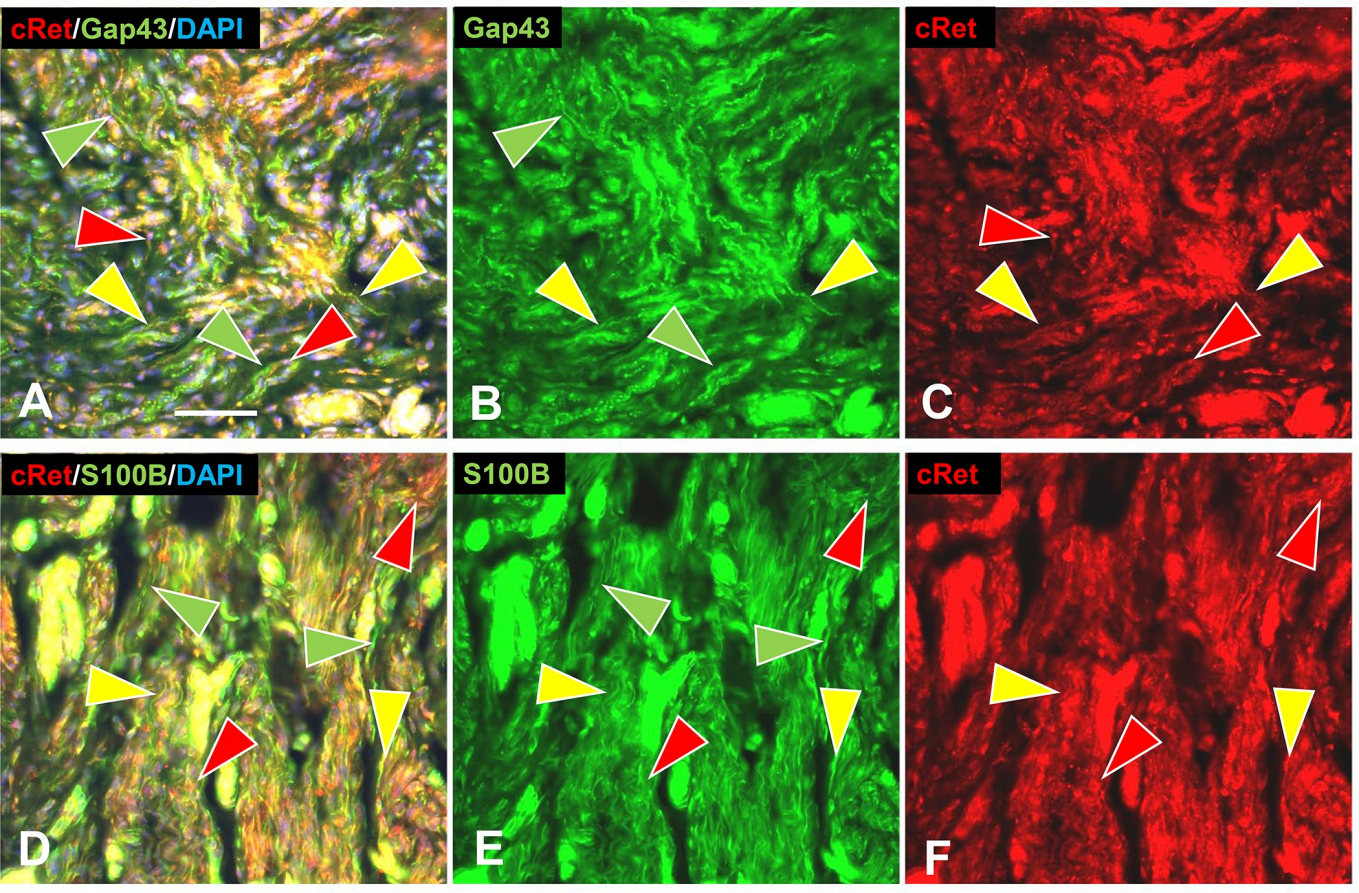
Multi-molecular immunolabeling with c-Ret. Immunolabeling for c-Ret is expressed on most of the aberrant innervation labeled for GAP-43 (A, B) and accompanying SC labeled for S100B (D, F) in s-cNF. A-C: Some nerve fibers labeled only for GAP-43 (green arrowheads), whereas others were double-labeled for both GAP-43 and c-Ret (yellow arrowheads). Other profiles labeled only for c-Ret (red arrowheads). D-F: Profiles were extensively co-labeled for c-Ret and S100B (yellow arrowheads). Fewer are labeled only for c-Ret (red arrowheads) or only for S100B (green arrowheads). Scale bar = 25μm.

**Fig 13:**
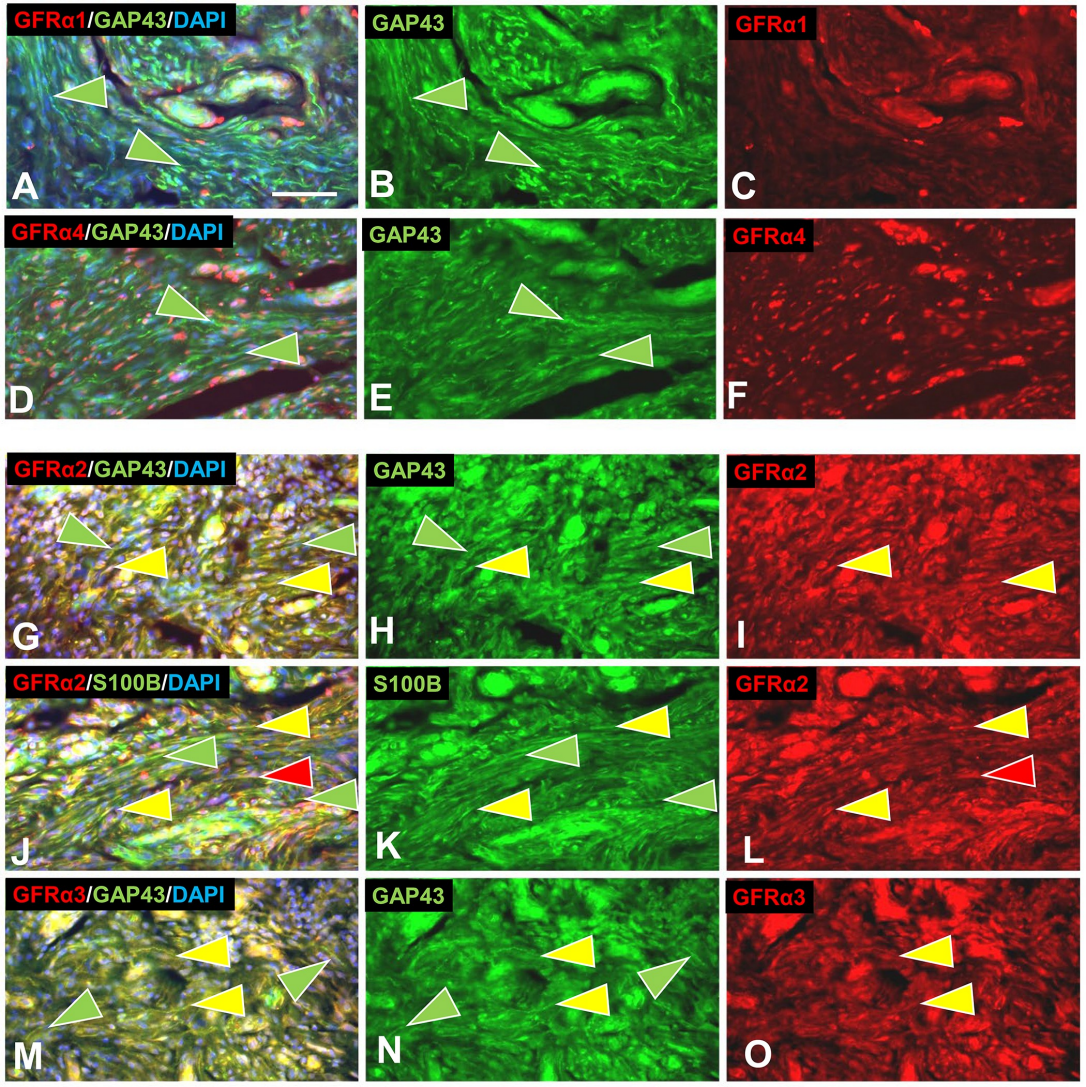
Aberrant innervation of s-cNF express co-receptors of GDNF family ligands. s-cNF aberrant innervation expresses immunolabeling for c-Ret co-receptors GFRα2 and GFRα3. A-F: No co-labeling was detected for GFRα1 and GFRα4 on the aberrant innervation labeled for GAP-43 (green arrowheads). G-L: Labeling for GFRα2 was widely co-expressed on or associated with most, but not all GAP-43-labeled aberrant innervation (yellow and green arrowheads). Co-labeling for S100B revealed some GFRα2 on SC as well as the innervation. M-O: GFRα3 is expressed on some, but not all of the GAP-43-labeled innervation (yellow and green arrowheads). GFRα3 was not obviously expressed on SC. Scale bar = 25μm.

Consistent with ITD-CMA indications that nonpeptidergic C-fiber were source of aberrant terminal sprouting in pre-cNF and s-cNF, immunolabeling for c-Ret was widely expressed not only on the aberrant innervation but also on the excessive nSC in the s-cNF (Fig 12A–12F), whereas immunolabeling for TrkA was inconclusive. Immunolabeling for c-Ret co-receptors in s-cNF failed to reveal GFRα1 and GFRα4, the primary co-receptors for GDNF and PSPN, respectively (Fig 13A–13F). Robust immunolabeling was detected for the NRTN co-receptor GFRα2 on both the aberrant innervation and nSC (Fig 13G–13L), and for the ARTN co-receptor GFRα3 primarily on the aberrant innervation (Fig 13M–13O).

Correspondingly, mRNA transcripts in s-cNF were consistently high for *RET* and *NTRN*, moderate to high for *GFRA2* and *GFRA3*, and moderate for *ARTN*. Transcripts were lower and more variable among s-cNF for *GDNF* and *PSPN*, and virtually non-existent for *GFRA1* and *GFRA4* (Fig 9). In comparison to normal skin, relative levels of transcripts in s-cNF were also significantly higher for *NRTN*, *GFRA2*, and *GFRA3*, comparable for *RET*, but significantly extremely lower for *ARTN* (Fig 9). *GFRA1* was significantly higher in s-cNF than in normal skin, but *GDNF*, *PSPN*, and *GFRA4* were significantly lower.

#### NGF family of neurotrophins

Consistent for inconclusive TrkA immunolabeling, transcripts for *NTRK1* ranged from low to virtually non-existent among s-cNF from different patients and was significantly relatively lower than in normal skin (Figs 9 and 10). Otherwise, among the various ligands and receptors of the NGF family, only *NTF4* transcripts were consistently high in s-cNF but its relative levels were significantly lower than in normal skin (Figs 9 and 10).

Transcripts were especially high for *NGFR* that translate to the low affinity nerve growth factor receptor (NGFR or p75) for all the NGF family ligands. Immunolabeling for NGFR in s-cNF was exceptionally robust, but surprisingly limited almost entirely to an unknown type of S100B negative cells (see Section D in S1 Text and S3 Fig) that seemingly forms partitions within the s-cNF like those described previously as compartmentalizing cells [30].

### Potential functional properties of aberrant s-cNF innervation

#### TrpA1 and TrpV1 detection

**Fig 14:**
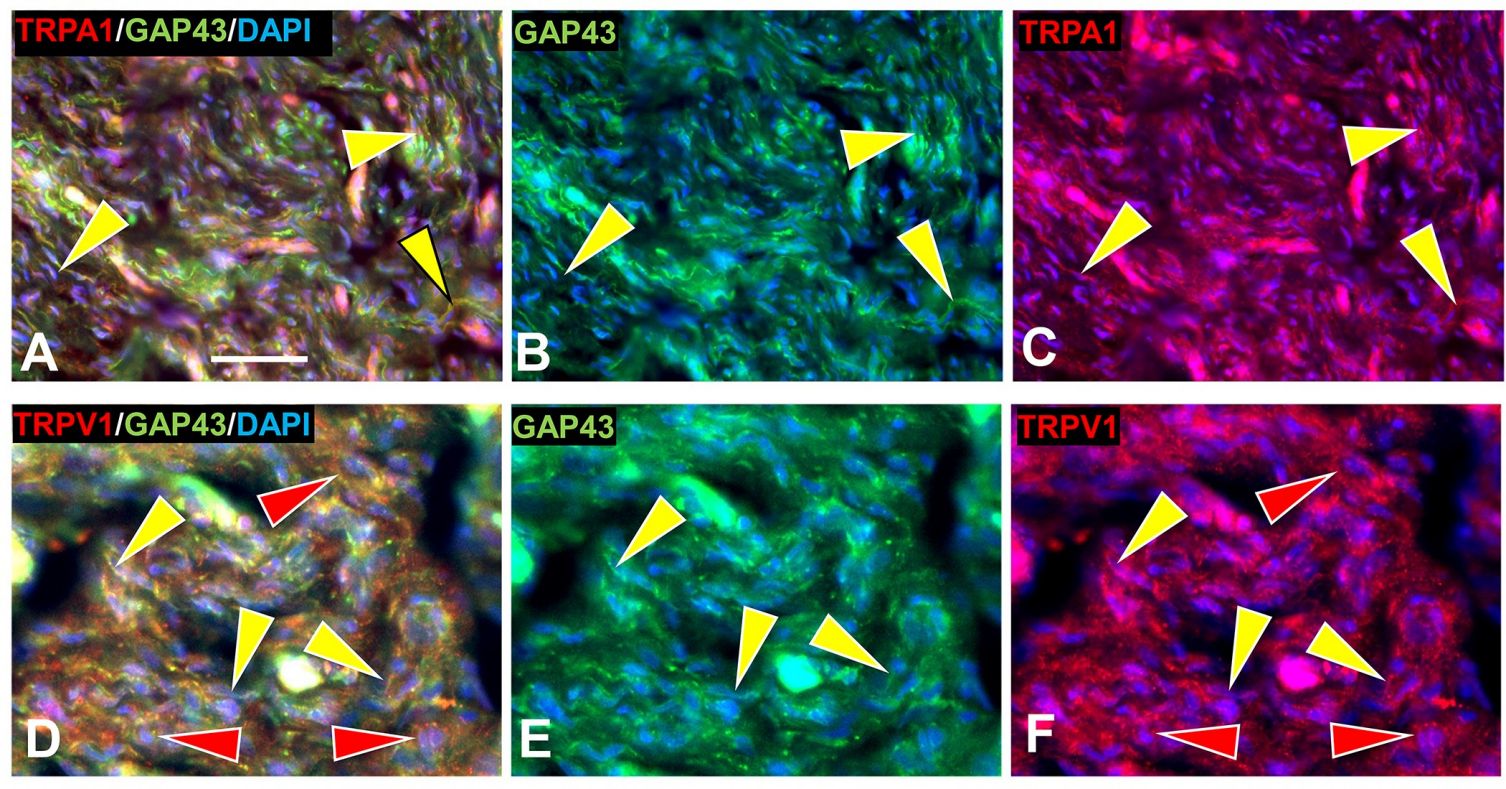
Immunolabeling for TrpA1 and TrpV1 in s-cNF. A. Immunolabeling in s-cNF of TrpA1 co-localized with GAP-43 on extensive aberrant innervation (yellow arrowheads). B. Immunolabeling in s-cNF of TrpV1 on at least some of the aberrant innervation (yellow arrowheads) but also on SC (red arrowheads). Scale bar = 25μm.

Previous studies demonstrated that ARTN/GFRα3/c-Ret signaling can enhance expression on nonpeptidergic C fibers of the cation channel transient receptor potential receptors, TrpA1 and TrpV1, which are implicated in itch and pain mechanisms, respectively [27, 29, 63, 84–89]. Immunolabeling of s-cNF detected extensive TrpA1 among the aberrant innervation (yellow arrowheads, Fig 14A–14C). TrpV1 immunolabeling was detected not only on at least some of the innervation but also among the associated SC (yellow and red arrowheads, respectively, Fig 14D–14F). Transcripts were consistently moderate for TrpA1 in s-cNF from different patients but relatively the same as in normal skin. However, consistent with TrpV1 immunolabeling on nSC, transcripts for *TRPV1* were consistently high among s-cNF and significantly higher relative transcripts in normal skin. Both TrpA1 and TrpV1 are known to be expressed in epidermal keratinocytes [89, 90], which would also be included among the assayed dissociated s-cNF components.

### Genetically-induced mouse pre-cNF

**Fig 15:**
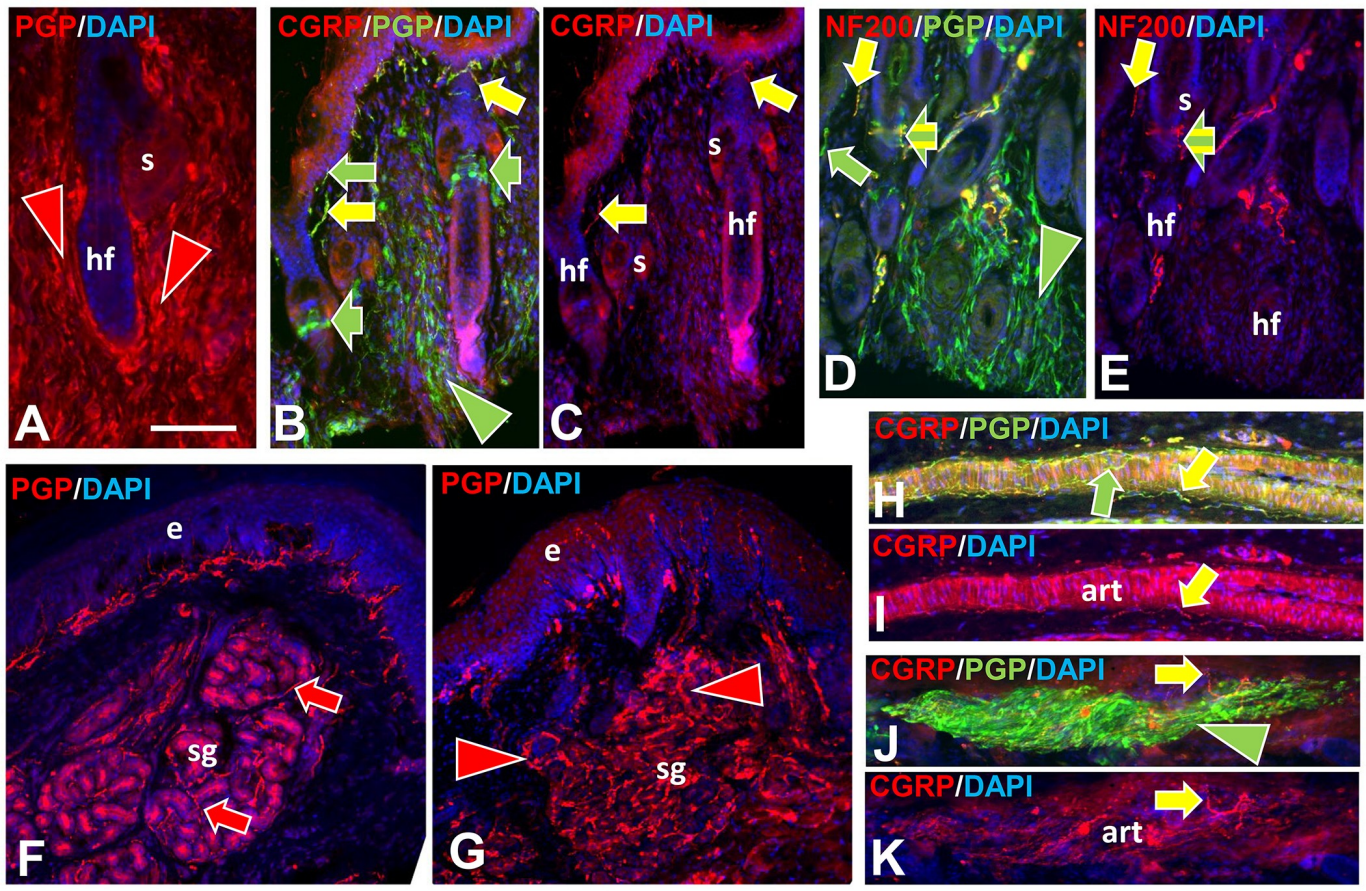
Immunolabeling among human-like pre-cNF in mice with SC-conditional *Nf1*/*Nf1* mutations. A-E: Hair follicles (hf) from the dorsum of the foot–some are engulfed in dense fine-caliber aberrant innervation (arrowheads). S = sebaceous gland. A. PGP labeling (red arrowheads) of innervation fully engulfing a follicle. B, C. Some follicles with normal innervation are shown to the left and a partially engulfed follicle to the right. Normal innervation with and without CGRP co-labeling of PGP is present in the upper dermis and neck of the hair follicle (yellow and green arrowheads), as well as normal pilo-neural innervation (broad arrows) located just below the follicle sebaceous glands. D, E. Some follicles with normal innervation are shown to the left and two follicles to the right partially in dense aberrant innervation (arrow head). Normal innervation is seen with and without NF200 (yellow and green arrows). The pilo-neural complexes have a mix of endings with and without NF200 (yellow and green striped arrows). F. Sweat gland with normal PGP-labeled innervation (red arrows). G. *Nf1/Nf1* sweat gland with dense aberrant innervation (red arrowheads). H, I. An arteriole with normal innervation labeled only for PGP (green arrow) and double-labeled for CGRP and PGP (yellow arrows). J.K. An *Nf1/Nf1* arteriole engulfed in dense aberrant innervation (green arrowhead), but with some normal CGRP innervation (yellow arrows). Scale bar = 50μm.

Human pNFs and cNF are thought to result from proliferation of SC that have undergone transformation from monoallelic to biallelic mutations of *NF1*. With our discovery of pre-cNF in humans, we re-investigated the mouse model developed previously by Wu et al., 2008 [21], which had a conditional SC-specific DI *Nf1* induced mutation. The original study revealed induced pNFs, but visible cNF were not evident. Consistent with indications that human cNF evolve from pre-cNF, our immunolabeling re-assessment of glabrous and hairy hind paw skin of these conditionally induced SC Nf1^-/-^ mice revealed pre-cNF-like pathologies associated with occasional hair follicles, sweat glands, and arterioles in two out of four mice (Fig 15). Consistent with the human pre-cNF, virtually all the excessive innervation lacked immunolabeling for CGRP, indicating it primarily consisted of nonpeptidergic C fibers. Overall, most hair follicles, sweat glands, and segments of arterioles had normal-appearing innervation in the PlpCre;Nf1^fl/fl^ mice indicating that the potential for triggering presumptive pre-cNF is increased but is nonetheless sporadic as in humans.

## Discussion

### Origin of cNF

The origin of pNF and cNF in NF1 patients has been attributed to an increased probability of aberrant hyperproliferation by nSC due to a nSC-specific spontaneous monoallelic to biallelic loss-of-function mutation of *NF1* resulting in a depletion of neurofibromin. Neurofibromin is normally implicated in a Ras-GTPase inhibitory modulation of several Ras-mediated functions that includes suppression of undesirable cell proliferation [15–18]. As a result of an increased probability for uncontrolled nSC proliferation, pNFs and cNF occur sporadically varying in frequency and size among NF1 patients [1–3, 8, 91, 92]. The NF1 mechanism is distinct from that in NF2 involving the mutation of *NF2*, which encodes the ezrin-radixin related protein, merlin, resulting in the development of true schwannomas rather than cNF or pNFs [93, 94]. The trigger for why suspected spontaneous SC biallelic mutations presumably occur and begin to hyperproliferate at random sites causing cNF is unknown [1, 6, 30].

### Adnexal pre-cNF as the site of cNF genesis

Although some innervation had been observed previously in cNF [30] and implicated in *NF1* DI SC hyperproliferation [95, 96], our ITD-CMA results of our study indicate that the cNF consistently originate among nonpeptidergic C-fiber terminals on adnexal skin structures: hair follicles, sweat glands, sweat ducts, sweat glands, arterioles, and AVS. Under normal conditions, each type of adnexal structure is innervated by structure-specific combinations of sensory and sympathetic terminals that are intimately in contact with terminal nSC [34, 36, 37, 49].

The implication of our ITD-CMA structural and molecular profiling is that each cNF begins in seemingly normal skin at an adnexal structure as a tiny microlesion (pre-cNF) that consists of a focal sprouting of sensory endings of nonpeptidergic C-fibers and a concomitant proliferation of their associated terminal nSC. As such, these aberrant terminals may contribute to chronic pain and itch in NF1 patients [1, 2, 22–25], which can occur in seemingly normal skin before the s-cNF appear. With the presumptive original adnexal structure consistently detected at the core, our ITD-CMA of s-cNF revealed that they have structural properties and neurochemical profiles consistent with a logical genesis from pre-cNF [34, 36, 37, 49] in that the nonpeptidergic C-fiber terminals and their associated nSC continue to sprout and proliferate to form the stalk and cap-like bulk of the emergent s-cNF. Once the process is initiated, the nSC continue to proliferate in excess of the sprouting innervation as the cNF increase in size. Consistent with terminal sprouting, virtually all of the aberrant innervation as revealed by anti-PGP-IL robustly co-expresses GAP43-IL (Section F in S1 Text).

Although molecular properties of innervation can deviate from normal under pathological conditions [32, 34, 53, 97–99], the ITD-CMA profile indicating that the sprouting consisted primarily of nonpeptidergic C-fiber terminals was surprising. Normally the nonpeptidergic C-fibers are just a small contingent among several other varieties of innervation that normally terminate on the various types of adnexal structures. For example, the predominant C-fiber innervation of each type of adnexal structure is peptidergic and normally expresses robust immunolabeling for CGRP, which was, at best, barely detectable among the aberrant innervation in the pre-cNF and s-cNF. Otherwise, additional dense unmyelinated innervation terminates on arterioles and AVS and on sweat glands that consists of noradrenergic and cholinergic sympathetic fibers, respectively, but none of their molecular properties were immunodetectable among the aberrant pre-cNF and s-cNF innervation. Finally, pre-cNF and s-cNF only contain sparse, if any definitive myelinated small-caliber Aδ fibers and large-caliber Aβ fibers that normally contribute some sensory endings on arterioles and AVS and several dense varieties of morphologically unique sensory endings on hair follicles. However, some of the s-cNF contained sparse Aβ fibers that were the source of unusual small, encapsulated endings that had been observed previously in cNF but have not been reported in normal human skin [100] (Section B in S1 Text).

### Nonmyelinating terminal SC, SC precursors, and other cell types in pre-cNF and s-cNF

#### Nonmyelinating SC

S100B labeling and the absence of MBP confirmed the presence of numerous mature SC intimately associated with the aberrant fibers both in pre-cNF and s-cNF. While C fibers and sympathetic fibers within nerves are normally sheathed only by nSC, all endings of sensory and sympathetic fibers are also normally intimately affiliated with specialized nSC, also referred to as terminal glia, regardless of whether the source axons are myelinated or unmyelinated. Within the pre-cNF and s-cNF, nSC at first appear to have proliferated in proportion to the excessive aberrant terminals but, in some s-cNF, there were locations where clusters of nSC apparently continued to proliferate in excess and independent of associated innervation.

#### Potential SC precursors

Both the pre-cNF and s-cNF contained clusters of cells that were S100B-negative but expressed TGFβ1, which has been implicated in promoting SC proliferation and differentiation especially under tumorigenic conditions [65–67, 88, 101–103]. In the s-cNF, they could be densely packed in zones intercalated with but segregated from zones containing the aberrant innervation and affiliated SC that were S100-positive but TGFβ1-negative. Both the TGFβ1 cells and mature nSC co-labeled for the transcription factor SOX-10, an SC developmental lineage marker [65, 69–72], thus indicating that the TGFβ1 cells were a potential precursor source of the excessive SC [6]. The functional genomics assessment of s-cNF confirmed a high level of message expression for TGFβ1 and SOX-10.

#### SKPs and BCCs

While it is known that SC are one of many cell types that are derived from neural crest cells [104–106], subtypes of SC exist that may have different lineages of differentiation, including from SKPs [107–111] and from BCCs [112–116]. The detection of SKPs to date has been limited primarily to the base of hair and whisker follicles in mice, which are sites that have little if any innervation and were not sites where pre-cNF were detected in NF1 patients.

By contrast, a line of mice that expresses a BCC-driven reporter gene revealed that BCC-derived nSC originate from BCCs located at the attachments of developing nerve roots to the spinal cord during early embryogenesis. BCC-derived SC migrate to developing nerve plexuses where pNFs evolve in NF1 patients, as well as through developing nerves to become terminal nSC in the skin where the pre-cNF are located [116]. Consistent with this observation, pNF were detected in our original assessment of an SC selective *Nf1* DI mouse model developed in 2008 [21]. Our reassessment, herein, revealed pre-cNF-like lesions in the skin of these mice that were located at various adnexa where the pre-cNF were discovered in our human NF1 patients. A recently developed BCC selective *Nf1* DI mouse model produced more obvious human-like cNF as well as pNFs [117]. This suggests that the hyperproliferating SC and likely TGFβ1-positive SC precursors in the pre-cNF and s-cNF in our NF1 patients arise *de novo* exclusively among the BCC-derived terminal SC. Whether or not they are *Nf1* DI remains to be determined.

Despite having different timings in their develop and potential to become malignant, an intriguing possibility is that the hyperproliferating SC in pNFs may also be a type of nonmyelinating terminal SC given that both pNFs and cNF have similar histological and functional genomics profiles and both may be derived from BCC lineages. In this case, the SC hyperproliferation in pNFs may involve terminal SC affiliated with arterioles within nerve plexuses that also have innervation, referred to as the *nervi nervorum*, like that of cutaneous arterioles [36, 118–121].

#### NGFR expressing “compartmentalizing” cells

Our ITD-CMA of the s-cNF failed to detect labeling for NGFR on the excessive nSC, as has been reported previously, despite high levels of NGFR message in the cNF. However, NGFR immunolabeling was intensely expressed among cells that were negative for S100B but seemed to form partitions among the aberrant innervation and excessive SC. Similar cells were described as compartmentalizing cells in a previous study. A definitive identity of these cells was not ascertained but may indicate a transition into another form of SC that may begin to infiltrate and become dominant in the further evolution of cNF.

#### Mast cells

Mast cells have been identified as a substantial contributor to cNF, possibly as a source of itch symptoms [27–29, 122]. Our histochemical assessment revealed more numerous but still relatively sparse distributions of mast cells in both the pre-cNF and s-cNF as compared to normal skin. Consequently, our impression is that mast cells and associated inflammatory mechanisms may be relatively minor factors in the initiation and early development of cNF [103], but may become more significant in later stages of cNF enlargement.

### Potential trophic mechanisms involved in the onset of pre-cNF and evolution into cNF

#### NTRN and ARTN signaling

Consistent with ITD-CMA indications that the aberrant innervation in pre-cNF and s-cNF consists primarily of terminals of nonpeptidergic C fibers, both the ITD-CMA and functional genomic analyses of the s-cNF indicated that NTRN and ARTN signaling mechanisms involving GFRα2 and GFRα3 may be interacting between the aberrant C-fiber terminals and associated nSC in initiating the pre-cNF and facilitating their evolution into s-cNF [86, 87].

The aberrant nonpeptidergic C fiber innervation in the s-cNF robustly immunolabeled for c-Ret and was inconclusive for TrkA. Additional immunolabeling combinations demonstrated co-labeling for the c-Ret co-receptor GFRα3 and likely some GFRα2, whose preferential signaling ligands are ARTN and NTRN, respectively. Both of these NTs are known modulators of primary afferent innervation and function [85, 88, 123]. Virtually no immunolabeling of the aberrant innervation was detected for GFRα1 and GFRα4, whose preferential signaling ligands are GDNF and PSPN, respectively.

Interestingly, the excessive associated nSC also robustly immunolabeled with c-Ret [124, 125], GFRα2, and perhaps some GFRα3, indicative of potential autocrine/paracrine signaling between the SC and between the aberrant innervation and SC [126–129]. Functional genomics assessments verified a consistently high level of *RET* and *NTRN* transcripts among s-cNF from different NF1 patients, as well as moderate to high levels for *GFRA2*, *GFRA3*, and *ARTN*. Compared to normal skin, relative transcripts for *NTRN*, *GFRA2*, and *GFRA3* were significantly higher in s-cNF whereas c-Ret was similar. Surprisingly, transcripts for *ARTN* were significantly lower in the cNF as compared to normal skin. Whereas Friedrich et al., 2016 ([30]) reported single immunolabeling for GDNF and GFRα1 as well as TrkA in cNF, our analyses detected little or was inconsistent for these or any of the ligands or other high affinity receptors at least among s-cNF.

Taken together, our results indicate that NTRN and ATRN signaling interactions between the C fiber terminals and their terminal SC, as well as autocrine/paracrine signaling among the SC, contribute to the initiation of pre-cNF and subsequent evolution into overt cNF. Consistent with a mutual interaction between the innervation and nSC in the onset and development of s-cNF, one report indicated that denervation of NF1 skin could reduce the number and size of cNF [130].

#### TrpA1 and TrpV1

Consistent with a known impact of ARTN and NTRN signaling on C fiber sensory neurons, the aberrant nonpeptidergic innervation in the s-cNF expressed immunolabeling for TrpA1 and TrpV1, which have previously been implicated in itch and pain mechanisms [29, 84, 86, 87, 89, 131–134]. As has been seen on other non-neuronal cells [89, 135], TrpV1 immunolabeling, supported by mRNA transcripts, was also detected on the excessive nSC in s-cNF, which had not been reported previously on normal SC. Therefore, the expression of TrpA1 and TrpV1 on the aberrant innervation and even TrpV1 on the excessive nSC in s-cNF may contribute to symptoms of itch and pain in NF1 patients.

#### What triggers the onset of pre-cNF and the evolution of cNF?

Our observations indicate that there is something about the nonpeptidergic C fiber terminals and their associated SC at a variety of adnexa that increases the probability of triggering a pre-cNF and their subsequent likely evolution into overt cNF. Strong evidence indicates that an *NF1-/-* transformation of SC is key to their hyperproliferation and genesis of all types of NF1 neurofibromas. Conditionally induced *Nf1* DI selectively in SC or BCCs of mice also produce neurofibromas within nerve plexuses and nerves as well as pre-cNF-like lesions in the skin, that seem to evolve into overt cNF in the BCCs of *Nf1* DI mice.

However, the various neurofibromas occur in *Nf1* DI mouse models in relatively few sites where mutated SC and BCCs are located. Likewise, while pre-cNF and s-cNF in NF1 patients apparently originate only among various types of cutaneous adnexal structures, they occur among relatively few of all possible adnexae even in patients with numerous cNF. Therefore, while *Nf1* DI transformations increase the probability that neurofibromas will occur, the mechanism(s) that actually triggers pNFs and cNF remains unknown.

Despite the considerable quantity of excessive innervation in the s-cNF, the likely origin from a small subset of sensory fibers associated with a specific adnexa suggests that relatively few DRG neurons may account for the excessive sprouting in each cNF. The capacity for excessive sprouting by C fibers has been shown in transgenic mouse lines designed to overproduce various neurotrophic factors in keratinocytes [76, 136].

Our observations indicate that the trigger for the occurrence of a pre-cNF and potential subsequent evolution into a cNF involves a local trophic interaction between nonpeptidergic C-fiber endings and their associated terminal SC at any type of adnexa that is a normal site where these endings are located. Moreover, the trophic interaction may involve ARTN- and/or NRTN- signaling through c-Ret expressed on both the nonpeptidergic C fibers and terminal SC. Whereas C fibers are typically regarded as nociceptors involved in pain sensation, there is increasing evidence that many, if not most, are normally involved in local homeostatic monitoring and maintenance of target structures.

The normal expression of GAP-43 among C fibers may be indicative of an ongoing remodeling of their sensory endings and associated terminal glia as part of a normal homeostatic monitoring subliminal maintenance which regularly fluctuates in response to transient tissue stressors, becoming especially exaggerated as part of wound repair [29, 35, 49, 113, 121, 137–141]. As such, the *NF1* germinal mutation may compromise the neural/SC cooperativity involved in normal tissue homeostasis. *NF1* DI may arise subsequently in an inappropriately mitotically active SC population [17]. Possibly, the adnexae themselves may exert some degree of paracrine tropism, specifically attracting critical cells from already-established NFs or other sites. Others have proposed possible seeding from mature neurofibromas and the existence of precursor lesions involving a specific neurofibroma precursor cell [8, 142]. Comparable to the NF1 pre-cNF, hamartia are microscopic lesions well-documented in the Tuberous Sclerosis Complex that precede and evolve into hamartomas [10].

## Conclusion

Our observations on the pre-cNF and s-cNF indicate that cNF are not initiated simply by autonomous clonal proliferations of SC or SC precursors that have undergone an *NF1*^*-/-*^ transformation. Rather, they appear to involve an initial trophic interaction between cutaneous C fiber sensory endings and their terminal SC associated with skin adnexae that may involve ARTN and NTRN signaling through c-Ret receptors on both the innervation and SC. This suggests that the pre-cNF and the evolution to cNF may arise on the basis of dysplasia [13] and not simply neoplasia in accordance with the canonical two-hit model. As such, therapeutic strategies that target the c-Ret signaling mechanism at the earliest appearance of a s-cNF may prevent the further development or maintenance of the cNF.

## Supporting information

S1 Text Supplemental text. (DOCX)

S1 Fig Secondary antibody labeling and autofluorescence. Secondary antibodies produced no detectable labeling above background autofluorescence in s-cNF. Example pairs of secondary antibody fluorescence in the absence of primary antibodies (A-E) as compared to autofluorescence (F). Note the autofluorescence of vascular profiles likely due to the presence of albumen (arrowheads). Scale bar = 50μm.
(TIF)

S2 Fig Mast cells (MC). Toluidine blue labeling revealed scattered, seemingly uniform distributions of MCs (arrowheads) in both a pre-cNF (A) and a s-cNF (B). Staining revealed MCs abnormally numerous and diffusely distributed throughout s-cNF (S2B Fig) as was known previously [19, 26, 63, 122]. Likewise, mast cells were diffusely distributed within the pre-cNF at a higher density than the surrounding dermis (S2A Fig). MC density was far less than that of TGFβ1 and of NRG-1 immunolabeled cells that also had a different morphology. Scale bar = 25μm.
(TIF)

S3 Fig Compartmentalizing Cells and Sensory Corpuscles. In s-cNF, NGFR immunolabeled cells (A-C, G-I; curved red arrows) have processes that form partitions among the aberrant innervation labeled for PGP (A-C, green arrowheads) and the accompanying S100B-labeled SC (G-I, curved green arrows and arrowheads). Some NGFR-positive cells appear to form enclosures (broad red arrows, A-C and G-I) of encapsulated large-caliber sensory endings that label for PGP (broad green arrows, A-C) and NF200 (broad yellow arrows, D-F). The matrix within the capsule and surrounding the ending labels with S100B and MBP (D-I). These are likely endings supplied by sparse large-caliber myelinated Aβ fibers that label for NF200 and MBP (large green arrows with red borders, D-F). Smaller-caliber lightly myelinated Aδ fibers are also present (small green arrows with red borders, D-F) seen losing their myelin sheath as it may be terminating (small green arrow). Note a capsule (broad red arrows) that contain a PGP-labeled large-caliber axon at the core (open yellow arrowheads). Relatively large-caliber axons also label for NGFR (solid yellow arrowheads, A-C) whereas the aberrant small-caliber axons lack NGFR (green arrowheads, A, B). Scale bar = 25μm.
(TIF)

## Abbreviations

AVS: Arteriole-Venule Shunt
BCC: Boundary Cap Cell
CGRP: Calcitonin Gene-Related Product
cNF: Cutaneous Neurofibroma
cRET: RET Proto-Oncogene
DAPI: 4’,6-diamidino-2-phenylindole, dihydrochloride
FNE: Free Nerve Ending
GAP-43: Growth-Associated Protein 43
GDNF: Glial-Derived Neurotrophic Factor
GFRα: Glial-Derived Neurotrophic Factor Family Receptor alpha
IF: Immunofluorescence
MBP: Myelin Basic Protein
MC: Mast Cell
nSC: nonmyelinating Schwann Cell
*Nf1*: the non-human mammalian gene/locus (italicized
NF1: the human gene/locus (italicized) and the syndrome (non-italicized)
NF200: 200kD neurofilament protein
NGF: Nerve Growth Factor
NGFR: Nerve Growth Factor Receptor, also referred to as p75
NRG-1: Neuregulin
NRTN: Neurturin
NSE: Neuron-Specific Enolase
pChAT: peripheral Choline-Acetyl Transferase
PGP: Protein Gene Product 9.5
pre-cNF: Cutaneous Pre-neurofibroma
PSPN: Persephin
SC: Schwann Cell
SKP: Skin-derived Precursor
TGFβ1: Transforming Growth Factor Beta 1
VAChT: Vesicular Acetylcholine Transporter
