# Supplementary material for: The evolution and multi-molecular properties of NF1 cutaneous neurofibromas originating from C-fiber sensory endings and terminal Schwann cells at normal sites of sensory terminations in the skin
Source: PLoS One. 2019 May 20;14(5):e0216527. doi: 10.1371/journal.pone.0216527 (PMC6527217; doi:10.1371/journal.pone.0216527)
Supplement: S1 Text — (DOCX) [file pone.0216527.s001.DOCX]

**S1 text: Supporting Information**

**Section A: Secondary antibody labeling and autofluorescence**

Secondary antibodies produced no detectable labeling above background autofluorescence in s-cNF (S1 Fig).

**Section B:** **NF200 expression in aberrant thin caliber innervation**

Although most of the aberrant GAP-43 expressing fibers in pre-cNF and the s-cNF has an immunolabeling profile consistent with sprouting from nonpeptidergic C-fiber terminals, a subset co-labeled for NF200, which is normally not expressed in mature C fibers and sympathetic fibers. Normally, NF200 is expressed on Aδ and Aβ fibers and their sensory endings along with MBP labeling of the myelin sheaths prior to terminating. Thus, the subset of aberrant NF200-fibers may be indicative of sprouting from Aδ and Aβ fibers [1-7]. However, NF200 is also expressed on all types of peripheral nerve fibers during development but normally down regulates in mature C fibers and sympathetic fibers and may be upregulating among many of the sprouting small fiber terminals.

**Section C: Mast cells**

Toluidine blue staining revealed MCs abnormally numerous and diffusely distributed throughout s-cNF (S2 Fig) as was known previously [8-11]. Likewise, mast cells were diffusely distributed within the pre-cNF at a higher density than the surrounding dermis (S2 Fig). Their density was far less than that of TGFβ1 and of NRG-1 immunolabeled cells that also had a different morphology.

**Section D:** **Compartmentalizing cells and sensory corpuscles**

Our mRNA profiling of s-cNF revealed high levels of expression for the low affinity NGFR (Fig. 8), which has been implicated in SC proliferation and reported on cells from NF1 neurofibromas [12], though others failed to detect it [13]. Therefore, we conducted a retrospective assessment of s-cNF sections and detected robust immunolabeling for NGFR on S100 negative cells with long processes organized into seemingly into thin partitions (S3 Fig A-C, G-I). This organization was consistent with what have previously been referred to as “compartmentalizing cells” by Friedrich et al. who did not find NGFR immunolabeling. They also formed capsules around small corpuscular endings seen by others in cNF [13-17], which are supplied by a large-caliber myelinated Aβ fibers (S3 Fig D-F). Unlike extremely large Pacinian corpuscles located deep in the dermis, such small corpuscular endings have rarely, if ever, been observed among the innervation to human or monkey keratinized skin but have been observed in the glabrous digital skin of raccoons [18]. Otherwise, s-cNF contained scattered small and large fibers that co-labeled for NF200 and MBP, indicative of Aδ and Aβ, that have low levels of NGFR immunoreactivity.

**Section E: Neurotrophins and receptors**

**NGF family**

The NGF family consists of the ligands nerve growth factor (NGF, gene *NGF*), brain-derived growth factor (BDNF, *BDNF*), neurotrophin-3 (NT3, *NTF3*), and neurotrophin-4 (NT4, *NTF4*) that signal through the high-affinity Trk family of receptor tyrosine kinases, TrkA (*NTRK1*), TrkB (*NTRK2*), and TrkC (*NTRK3*) [19-21]. NGF/TrkA signaling is required for all types of C fibers during development, axon targeting, and early survival, and for continued maintenance of peptidergic C fibers into adulthood.

**GDNF family**

The GDNF family consists of the ligands GDNF (*GDNF*), neurturin (NRTN, *NRTN*), artemin (ARTN, *ARTN*), and persephin (PSPN, *PSPN*) that signal through a common tyrosine-kinase receptor (cRET, *RET*) by means of separate co-receptors, GFRα1-4 (*GFRA1-4*) [22-27]. Whereas, both peptidergic and nonpeptidergic C fibers depend up NGF/TRKA signaling for their initial development, nonpeptidergic C fibers switch to a dependency on cRET signaling for further development [22-24, 28].

**Section F: GAP-43 expression in sprouting innervation**

Consistent with the assumption that terminals of nonpeptidergic C fibers are sprouting, virtually all of the aberrant innervation in the pre-cNF and s-cNF labeled for the axon growth marker GAP-43, which is normally expressed in all types of cutaneous sensory and autonomic innervation during development. However, the expression of GAP-43 is not uniquely indicative of aberrant, *de novo* sprouting because it normally continues to be expressed in nearly all types of mature C-fibers and sympathetic fibers after they terminate at their targets, whereas GAP-43 normally is downregulated in mature Aδ and Aβ fibers [1, 3]. Furthermore, the terminals of most types of C fibers and sympathetic fibers often have a simple branched or unbranched morphology referred to as FNE, whereas different types of myelinated sensory fibers typically terminate as a variety of morphologically distinctive endings, such as lanceolate endings around hair follicles, Meissner corpuscles in dermal papillae, corpuscular endings in the dermis, or Merkel endings on Merkel cells in the basal layer of the epidermis. However, under pathological conditions, the morphology and distribution of Aδ and Aβ can reorganize accompanied by an upregulation of GAP-43 [1, 3].

**Supporting Information References**

1. Albrecht PJ, Hines S, Eisenberg E, Pud D, Finlay DR, Connolly MK, et al. Pathologic alterations of cutaneous innervation and vasculature in affected limbs from patients with complex regional pain syndrome. Pain. 2006;120(3):244-66. PubMed PMID: 16427199.

2. Carriel V, Garzon I, Campos A, Cornelissen M, Alaminos M. Differential expression of GAP-43 and neurofilament during peripheral nerve regeneration through bio-artificial conduits. J Tissue Eng Regen Med. 2017;11(2):553-63. Epub 2014/08/02. doi: 10.1002/term.1949. PubMed PMID: 25080900.

3. Pare M, Albrecht PJ, Noto CJ, Bodkin NL, Pittenger GL, Schreyer DJ, et al. Differential hypertrophy and atrophy among all types of cutaneous innervation in the glabrous skin of the monkey hand during aging and naturally occurring type 2 diabetes. J Comp Neurol. 2007;501(4):543-67. PubMed PMID: 17278131.

4. Boumil E, Vohnoutka R, Lee S, Shea TB. Early expression of the high molecular weight neurofilament subunit attenuates axonal neurite outgrowth. Neurosci Lett. 2015;604:36-41. Epub 2015/08/01. doi: 10.1016/j.neulet.2015.07.031 S0304-3940(15)30051-3 [pii]. PubMed PMID: 26225928.

5. Yuan A, Sasaki T, Kumar A, Peterhoff CM, Rao MV, Liem RK, et al. Peripherin is a subunit of peripheral nerve neurofilaments: implications for differential vulnerability of CNS and peripheral nervous system axons. J Neurosci. 2012;32(25):8501-8. Epub 2012/06/23. doi: 10.1523/JNEUROSCI.1081-12.2012 32/25/8501 [pii]. PubMed PMID: 22723690; PubMed Central PMCID: PMC3405552.

6. Fricker FR, Zhu N, Tsantoulas C, Abrahamsen B, Nassar MA, Thakur M, et al. Sensory axon-derived neuregulin-1 is required for axoglial signaling and normal sensory function but not for long-term axon maintenance. J Neurosci. 2009;29(24):7667-78. Epub 2009/06/19. doi: 10.1523/JNEUROSCI.6053-08.2009 29/24/7667 [pii]. PubMed PMID: 19535578; PubMed Central PMCID: PMC2875847.

7. Walker KL, Yoo HK, Undamatla J, Szaro BG. Loss of neurofilaments alters axonal growth dynamics. J Neurosci. 2001;21(24):9655-66. Epub 2001/12/12. doi: 21/24/9655 [pii]. PubMed PMID: 11739575.

8. Liao CP, R CB, Brosseau JP, Chen Z, Mo J, Tchegnon E, et al. Contributions of inflammation and tumor microenvironment to neurofibroma tumorigenesis. J Clin Invest. 2018. Epub 2018/03/30. doi: 10.1172/JCI99424 99424 [pii]. PubMed PMID: 29596064.

9. Baratelli F, Le M, Gershman GB, French SW. Do mast cells play a pathogenetic role in neurofibromatosis type 1 and ulcerative colitis? Exp Mol Pathol. 2014;96(2):230-4. Epub 2014/03/04. doi: 10.1016/j.yexmp.2014.02.006 S0014-4800(14)00021-5 [pii]. PubMed PMID: 24583366.

10. Tucker T, Riccardi VM, Sutcliffe M, Vielkind J, Wechsler J, Wolkenstein P, et al. Different patterns of mast cells distinguish diffuse from encapsulated neurofibromas in patients with neurofibromatosis 1. J Histochem Cytochem. 2011;59(6):584-90. Epub 2011/04/29. doi: 10.1369/0022155411407340 0022155411407340 [pii]. PubMed PMID: 21525187; PubMed Central PMCID: PMC3201189.

11. Yang FC, Ingram DA, Chen S, Hingtgen CM, Ratner N, Monk KR, et al. Neurofibromin-deficient Schwann cells secrete a potent migratory stimulus for Nf1+/- mast cells. J Clin Invest. 2003;112(12):1851-61. Epub 2003/12/18. doi: 10.1172/JCI19195 112/12/1851 [pii]. PubMed PMID: 14679180; PubMed Central PMCID: PMC296994.

12. Yamamoto M, Sobue G, Li M, Arakawa Y, Mitsuma T, Kimata K. Nerve growth factor (NGF), brain-derived neurotrophic factor (BDNF) and low-affinity nerve growth factor receptor (LNGFR) mRNA levels in cultured rat Schwann cells; differential time- and dose-dependent regulation by cAMP. Neurosci Lett. 1993;152(1-2):37-40. Epub 1993/04/02. doi: 0304-3940(93)90477-3 [pii]. PubMed PMID: 8390628.

13. Friedrich RE, Holstein AF, Middendorff R, Davidoff MS. Vascular wall cells contribute to tumourigenesis in cutaneous neurofibromas of patients with neurofibromatosis type 1. A comparative histological, ultrastructural and immunohistochemical study. Anticancer Res. 2012;32(5):2139-58. Epub 2012/05/18. doi: 32/5/2139 [pii]. PubMed PMID: 22593502.

14. Onishi A, Nada O. Ultrastructure of the onion bulb-like lamellated structure observed in the sural nerve in a case of von Recklinghausen's disease. Acta Neuropathol. 1972;20(3):258-63. Epub 1972/01/01. PubMed PMID: 4625499.

15. Watabe K, Kumanishi T, Ikuta F, Oyake Y. Tactile-like corpuscles in neurofibromas: immunohistochemical demonstration of S-100 protein. Acta Neuropathol. 1983;61(3-4):173-7. Epub 1983/01/01. PubMed PMID: 6359808.

16. Weiser G. An electron microscope study of "Pacinian neurofibroma". Virchows Arch A Pathol Anat Histol. 1975;366(4):331-40. Epub 1975/01/01. PubMed PMID: 808023.

17. Albuerne M, Lopez S, Naves FJ, Martinez-Almagro A, Represa J, Vega JA. S100alpha and S100beta proteins in human cutaneous sensory corpuscles: effects of nerve and spinal cord injury. The Anatomical record. 1998;251(3):351-9. Epub 1998/07/21. doi: 10.1002/(SICI)1097-0185(199807)251:3<351::AID-AR11>3.0.CO;2-N [pii]. PubMed PMID: 9669763.

18. Rice FL, Rasmusson DD. Innervation of the digit on the forepaw of the raccoon. J Comp Neurol. 2000;417(4):467-90. Epub 2000/03/04. doi: 10.1002/(SICI)1096-9861(20000221)417:4<467::AID-CNE6>3.0.CO;2-Q [pii]. PubMed PMID: 10701867.

19. Markus A, Patel TD, Snider WD. Neurotrophic factors and axonal growth. Curr Opin Neurobiol. 2002;12(5):523-31. Epub 2002/10/09. doi: S0959438802003720 [pii]. PubMed PMID: 12367631.

20. Rice FL, Albers KM, Davis BM, Silos-Santiago I, Wilkinson GA, LeMaster AM, et al. Differential dependency of unmyelinated and A delta epidermal and upper dermal innervation on neurotrophins, trk receptors, and p75LNGFR. Dev Biol. 1998;198(1):57-81. Epub 1998/06/26. doi: S0012160698988613 [pii]. PubMed PMID: 9640332.

21. Patapoutian A, Reichardt LF. Trk receptors: mediators of neurotrophin action. Curr Opin Neurobiol. 2001;11(3):272-80. Epub 2001/06/12. doi: S0959-4388(00)00208-7 [pii]. PubMed PMID: 11399424.

22. Airaksinen MS, Saarma M. The GDNF family: signalling, biological functions and therapeutic value. Nat Rev Neurosci. 2002;3(5):383-94. Epub 2002/05/04. doi: 10.1038/nrn812 nrn812 [pii]. PubMed PMID: 11988777.

23. Fundin BT, Mikaels A, Westphal H, Ernfors P. A rapid and dynamic regulation of GDNF-family ligands and receptors correlate with the developmental dependency of cutaneous sensory innervation. Development. 1999;126(12):2597-610. Epub 1999/05/20. PubMed PMID: 10331972.

24. Baloh RH, Enomoto H, Johnson EM, Jr., Milbrandt J. The GDNF family ligands and receptors - implications for neural development. Curr Opin Neurobiol. 2000;10(1):103-10. Epub 2000/02/19. doi: S0959-4388(99)00048-3 [pii]. PubMed PMID: 10679429.

25. Milbrandt J, de Sauvage FJ, Fahrner TJ, Baloh RH, Leitner ML, Tansey MG, et al. Persephin, a novel neurotrophic factor related to GDNF and neurturin. Neuron. 1998;20(2):245-53. Epub 1998/03/10. doi: S0896-6273(00)80453-5 [pii]. PubMed PMID: 9491986.

26. Neet KE, Campenot RB. Receptor binding, internalization, and retrograde transport of neurotrophic factors. Cell Mol Life Sci. 2001;58(8):1021-35. Epub 2001/09/01. doi: 10.1007/PL00000917 [pii] 10.1007/PL00000917. PubMed PMID: 11529495.

27. Wang X. Structural studies of GDNF family ligands with their receptors-Insights into ligand recognition and activation of receptor tyrosine kinase RET. Biochim Biophys Acta. 2013;1834(10):2205-12. Epub 2012/10/23. doi: 10.1016/j.bbapap.2012.10.008 S1570-9639(12)00249-X [pii]. PubMed PMID: 23085183.

28. Molliver DC, Wright DE, Leitner ML, Parsadanian AS, Doster K, Wen D, et al. IB4-binding DRG neurons switch from NGF to GDNF dependence in early postnatal life. Neuron. 1997;19(4):849-61. Epub 1997/11/14. doi: S0896-6273(00)80966-6 [pii]. PubMed PMID: 9354331.
