# Supplementary figures and images for: The evolution and multi-molecular properties of NF1 cutaneous neurofibromas originating from C-fiber sensory endings and terminal Schwann cells at normal sites of sensory terminations in the skin
Source: PLoS One. 2019 May 20;14(5):e0216527. doi: 10.1371/journal.pone.0216527 (PMC6527217; doi:10.1371/journal.pone.0216527)

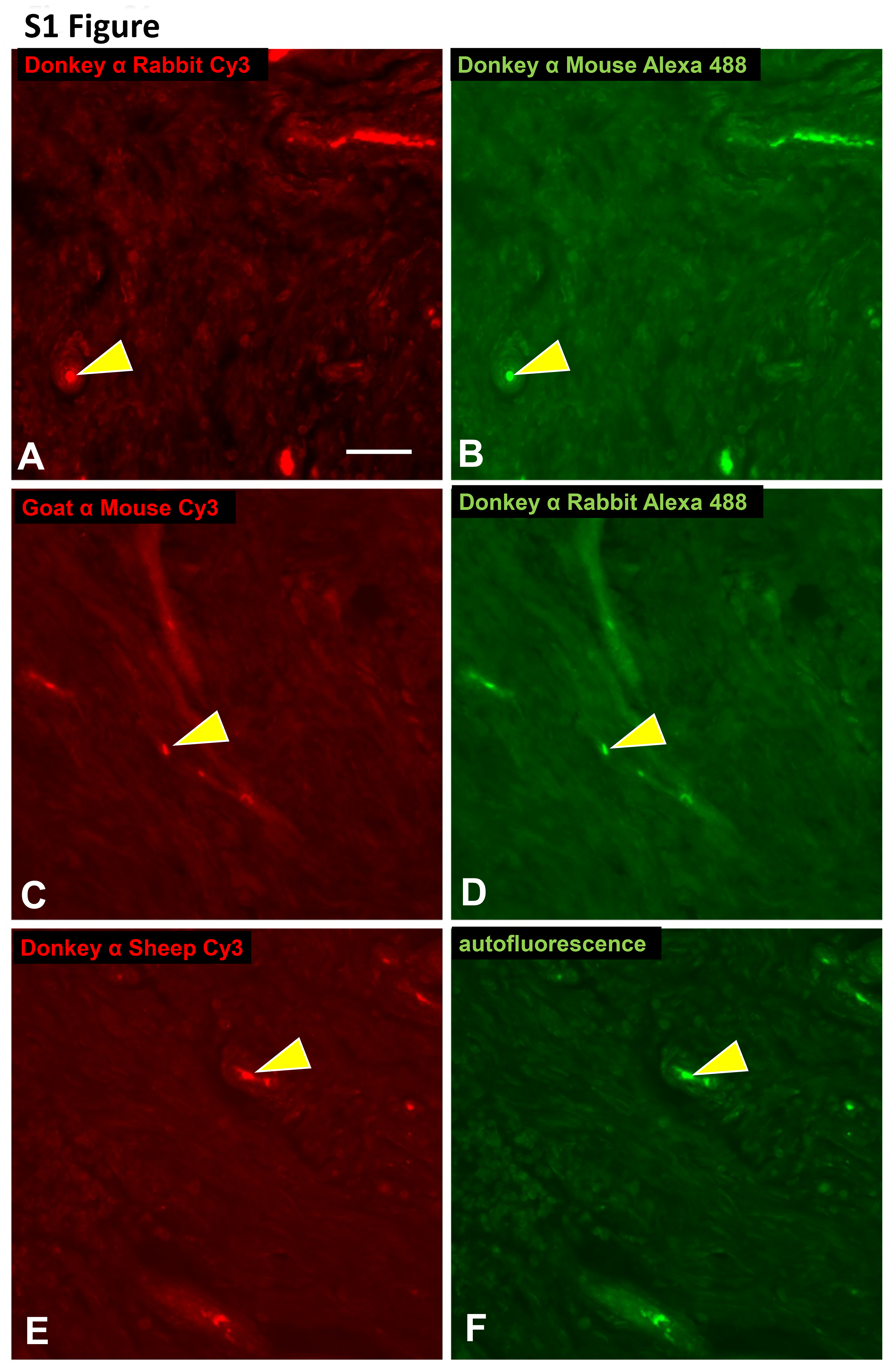

Supplement: S1 Fig — Secondary antibodies produced no detectable labeling above background autofluorescence in s-cNF. Example pairs of secondary antibody fluorescence in the absence of primary antibodies (A-E) as compared to autofluorescence (F). Note the autofluorescence of vascular profiles likely due to the presence of albumen (arrowheads). Scale bar = 50μm. (TIF) [file pone.0216527.s002.tif]

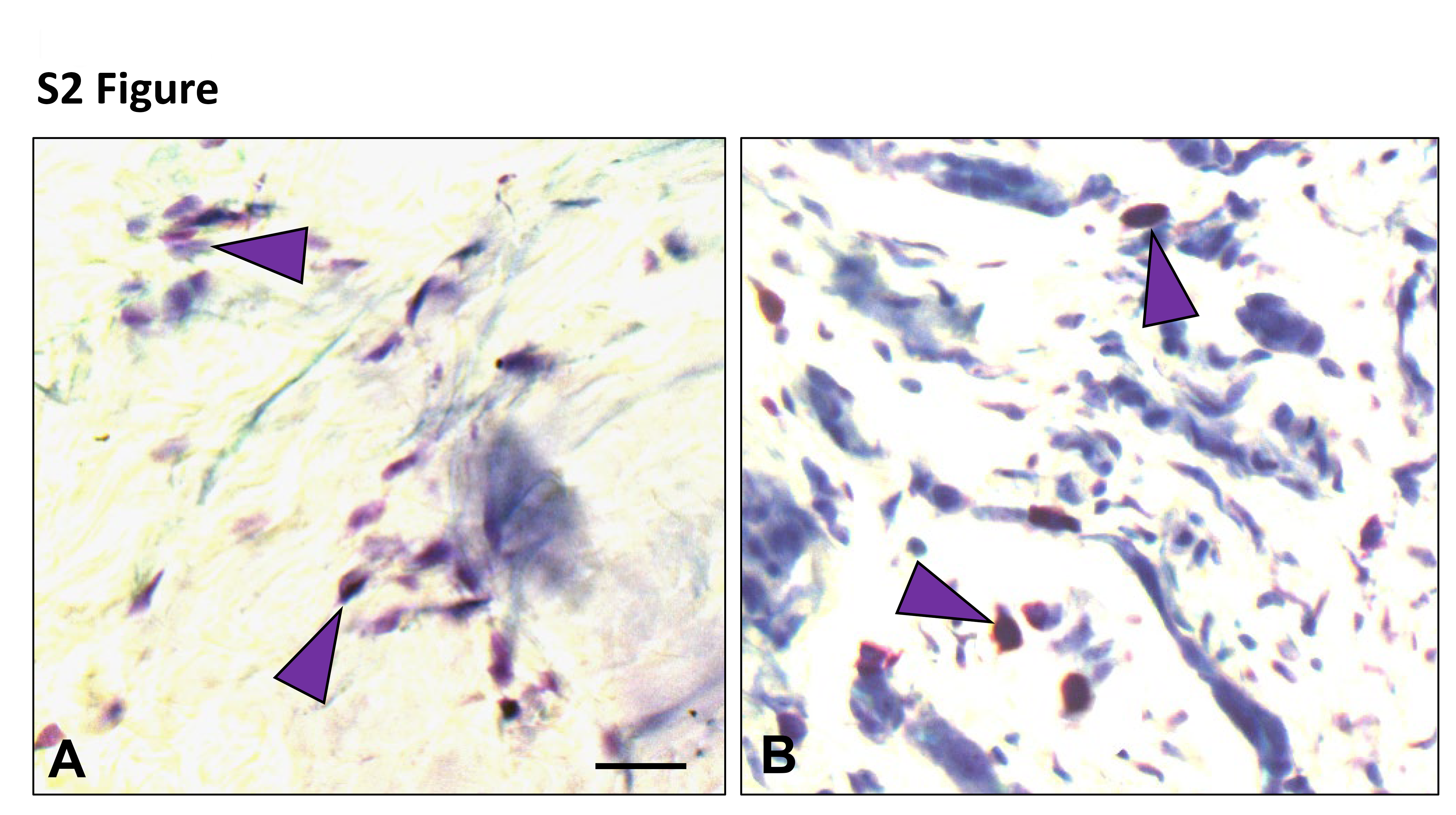

Supplement: S2 Fig — Toluidine blue labeling revealed scattered, seemingly uniform distributions of MCs (arrowheads) in both a pre-cNF (A) and a s-cNF (B). Staining revealed MCs abnormally numerous and diffusely distributed throughout s-cNF (S2B Fig) as was known previously [19, 26, 63, 122]. Likewise, mast cells were diffusely distributed within the pre-cNF at a higher density than the surrounding dermis (S2A Fig). MC density was far less than that of TGFβ1 and of NRG-1 immunolabeled cells that also had a different morphology. Scale bar = 25μm. (TIF) [file pone.0216527.s003.tif]

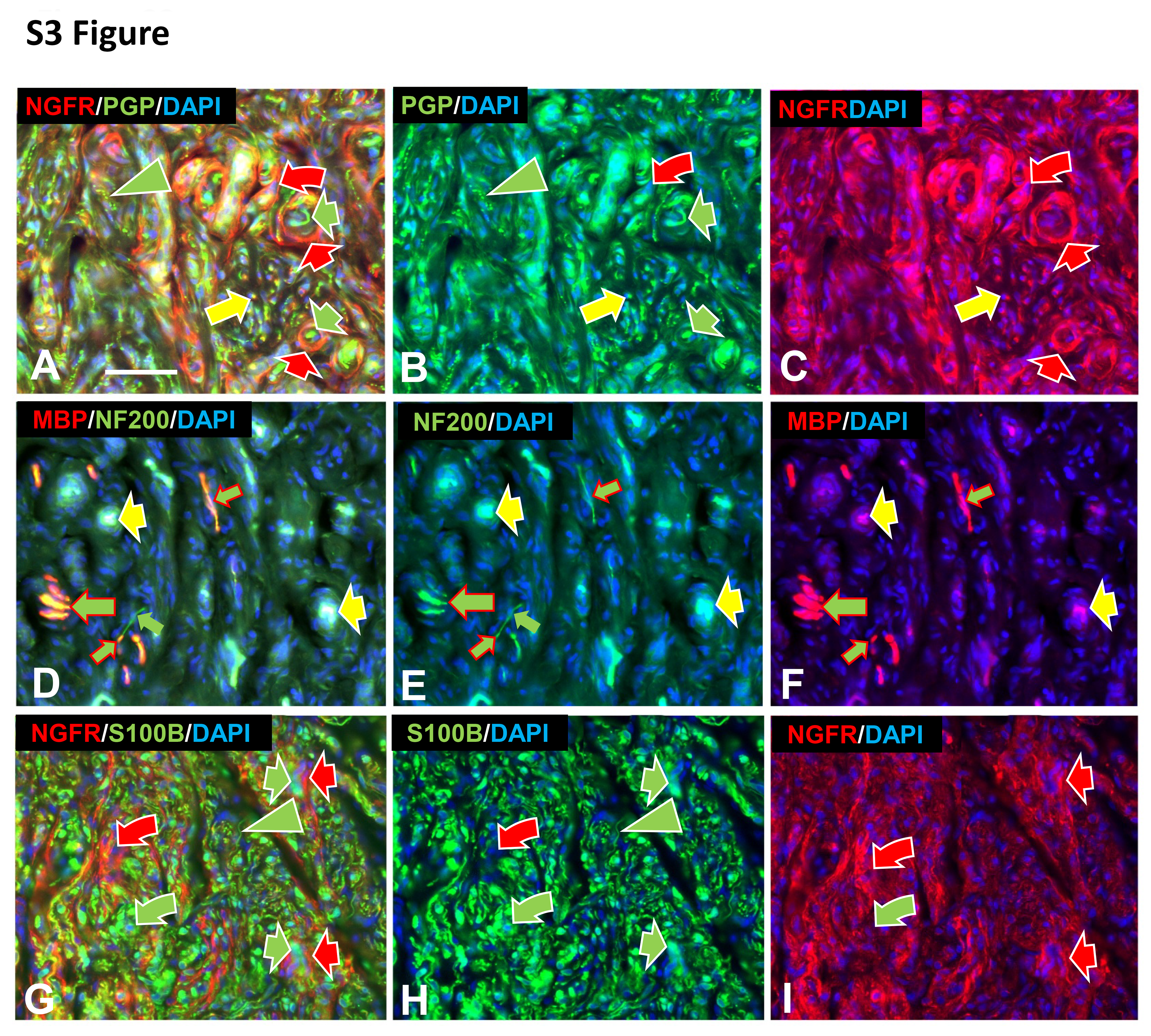

Supplement: S3 Fig — In s-cNF, NGFR immunolabeled cells (A-C, G-I; curved red arrows) have processes that form partitions among the aberrant innervation labeled for PGP (A-C, green arrowheads) and the accompanying S100B-labeled SC (G-I, curved green arrows and arrowheads). Some NGFR-positive cells appear to form enclosures (broad red arrows, A-C and G-I) of encapsulated large-caliber sensory endings that label for PGP (broad green arrows, A-C) and NF200 (broad yellow arrows, D-F). The matrix within the capsule and surrounding the ending labels with S100B and MBP (D-I). These are likely endings supplied by sparse large-caliber myelinated Aβ fibers that label for NF200 and MBP (large green arrows with red borders, D-F). Smaller-caliber lightly myelinated Aδ fibers are also present (small green arrows with red borders, D-F) seen losing their myelin sheath as it may be terminating (small green arrow). Note a capsule (broad red arrows) that contain a PGP-labeled large-caliber axon at the core (open yellow arrowheads). Relatively large-caliber axons also label for NGFR (solid yellow arrowheads, A-C) whereas the aberrant small-caliber axons lack NGFR (green arrowheads, A, B). Scale bar = 25μm. (TIF) [file pone.0216527.s004.tif]
